# Release of cognitive and multimodal MRI data including real-world tasks and hippocampal subfield segmentations

**DOI:** 10.1038/s41597-023-02449-9

**Published:** 2023-08-16

**Authors:** Ian A. Clark, Eleanor A. Maguire

**Affiliations:** Wellcome Centre for Human Neuroimaging, Department of Imaging Neuroscience, UCL Queen Square Institute of Neurology, University College London, London, UK

**Keywords:** Cognitive neuroscience, Learning and memory

## Abstract

We share data from N = 217 healthy adults (mean age 29 years, range 20–41; 109 females, 108 males) who underwent extensive cognitive assessment and neuroimaging to examine the neural basis of individual differences, with a particular focus on a brain structure called the hippocampus. Cognitive data were collected using a wide array of questionnaires, naturalistic tests that examined imagination, autobiographical memory recall and spatial navigation, traditional laboratory-based tests such as recalling word pairs, and comprehensive characterisation of the strategies used to perform the cognitive tests. 3 Tesla MRI data were also acquired and include multi-parameter mapping to examine tissue microstructure, diffusion-weighted MRI, T2-weighted high-resolution partial volume structural MRI scans (with the masks of hippocampal subfields manually segmented from these scans), whole brain resting state functional MRI scans and partial volume high resolution resting state functional MRI scans. This rich dataset will be of value to cognitive and clinical neuroscientists researching individual differences, real-world cognition, brain-behaviour associations, hippocampal subfields and more. All data are freely available on Dryad.

## Background & Summary

The genesis of the dataset we are sharing here was the desire to examine a brain structure called the hippocampus in order to learn more about individual differences in its structure and function among healthy adult humans. Our particular interest was in real-world cognition, and how the hippocampus supports functions such as the ability to imagine naturalistic scenes and events including those that might happen in the future, autobiographical memory – the memory of our past experiences, and spatial navigation. Decades of work involving human participants and rodents has implicated the hippocampus in these critical aspects of cognition^1–6^, and damage to the hippocampus can devastate these abilities^7–13^.

In order to properly assess individual differences in real-world cognition and the associations, if any, with the hippocampus, the brain scans of at least several hundred participants are required^14,15^, accompanied by a wide variance in cognitive test performance. Numerous large open access databases exist that contain magnetic resonance imaging (MRI) scans or magnetoencephalography (MEG) data along with scores from cognitive tests including those assessing memory, such as recognition memory for single words^16,17^. However, there is a dearth of databases that include tests of real-world cognition. Perhaps the most relevant are those that contain neuroimaging data acquired while participants passively watched movies or television programmes, which is more naturalistic [e.g.^18–21^, see also^22^]. However, large datasets that have neuroimaging data along with tests tapping into people’s actual lived experience are few – for an exception see^23,24^, which includes MRI scans and data from an autobiographical memory recall test. Examining real-world cognition is important because it has been shown, for example, that there are different neural substrates associated with autobiographical memory retrieval and the recall of laboratory-based stimuli^25^. Moreover, there is increasing awareness across cognitive neuroscience that brains do not live or go awry in laboratories, but rather in the multidimensional, ever-changing real world that is difficult to replicate in laboratory-based tasks^26–32^. We acknowledge that what we have called here “real-world tests” did not involve data collected in the field, so to speak. However, autobiographical memory recall and navigation are highly reflective of lived experiences. Similarly, most of us engage in the imagination of scenes and events particularly in the service of future thinking. Consequently, we use the term “real-world tests” for these cognitive tasks, as they stand in clear contrast to tests involving much simpler or more abstracted stimuli that are typically found in laboratory-based experiments.

Another absentee from databases is information about how participants perform cognitive tests. The use of cognitive strategies during simpler, laboratory-based tests, such as word list learning, has been studied extensively. In this domain, strategies have been found to differ in terms of their modality, including visual imagery and verbal strategies involving sentences or stories, and in their complexity, ranging from simple strategies like rote repetition to more complex strategies involving bizarre and distinct visual imagery and interactive visual scenes^33–39^. By contrast, the strategies people use to perform tests assessing real-world cognition have been under-studied. Moreover, cognitive strategy information relating to laboratory-based or real-world tests is not available in any large open access database, to the best of our knowledge. This is unfortunate because such information could provide another perspective on cognition, and augment our understanding of the cognitive processes involved when performing tests, which could aid in the interpretation of results.

**Fig. 1:**
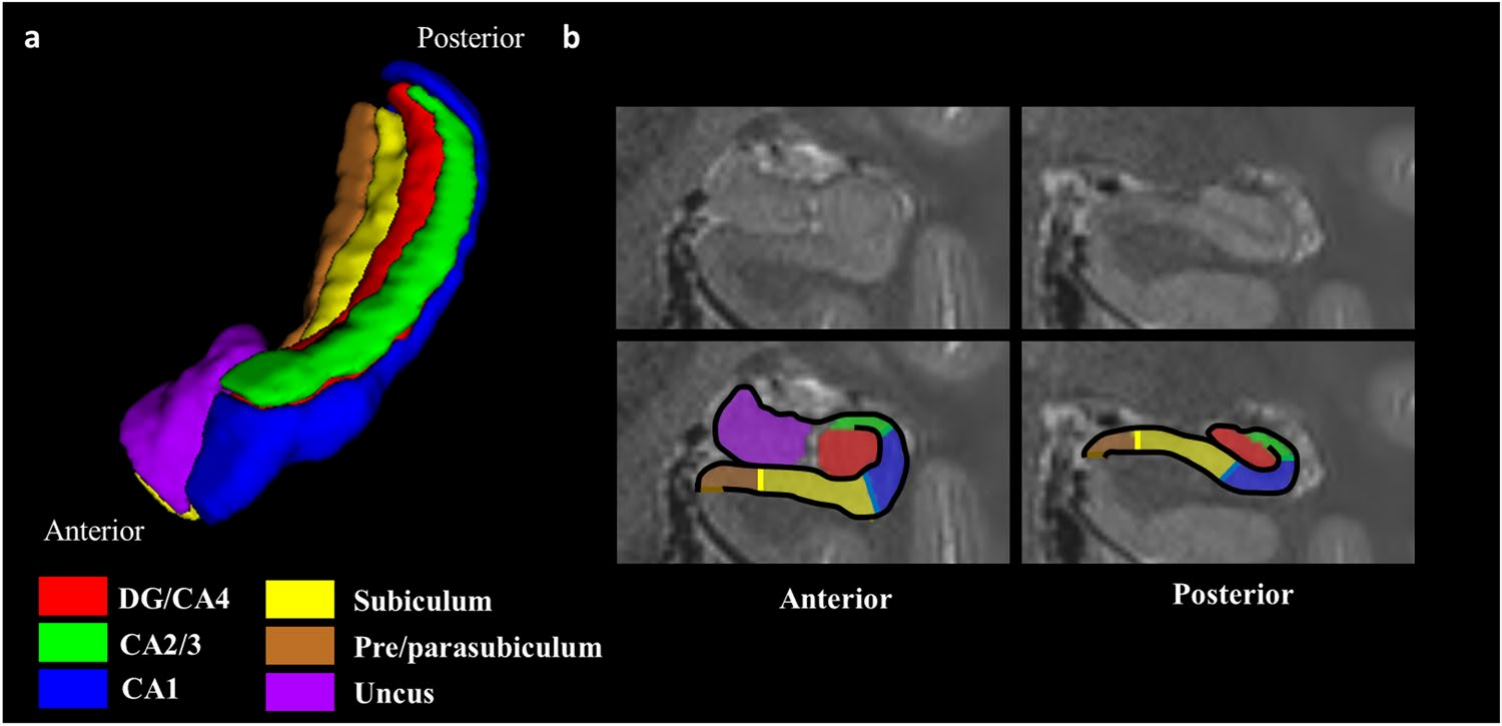
The hippocampus and its subregions. (**a**) A 3D representation, viewed from an antero-lateral perspective, of the segmented hippocampal subregions. (**b**) Two sections from T2-weighted structural MRI scans of an example hippocampus (top panel), overlaid with hippocampal subfield masks (lower panel). The left image is from the anterior hippocampus, and the right image from the posterior hippocampus. DG = dentate gyrus, CA = Cornu Ammonis.

So large open access datasets containing neuroimaging data, scores from real-world tests and information about the cognitive strategies deployed are lacking. The picture is made more complex by the fact that the hippocampus is not a homongeneous brain structure (see Fig. 1). It comprises anatomically distinct subregions – the dentate gyrus (DG), Cornu Ammonis (CA) 1–4, the subiculum, the presubiculum and parasubiculum (often studied together in humans as one region called the pre/parasubiculum) and an anatomically complex region called the uncus^40–42^. Each of these subfields has different connections to other brain areas^43–49^. Little is known about precisely how cognitive processes such as imagination, autobiographical memory and spatial navigation map on to the subfields in humans. The small number of studies there are have linked CA3 to autobiographical memory recall^9,50–52^, and the pre/parasubiculum to the imagination of the scenes and events that might underpin autobiographical memory and future thinking^53–56^.

Despite subfields being the key to understanding hippocampal function, the investigations into human hippocampal subfields are still relatively few and typically have small sample sizes because it is challenging to delineate subfields from structural MRI scans. While automated methods for subfield segmentation are available [e.g.^57^], the considerable inter-subject variability in the morphology of the hippocampus makes it difficult to achieve sufficient accuracy, especially along the full length of the hippocampus. Consequently, manual delineation of hippocampal subfields remains the gold standard^58^. This requires expertise and is time-consuming (~8 hours per participant when performed by an experienced segmenter), especially if performed at scale. Consequently, no large open access database includes six manually segmented subfields along the entire length of the hippocampus for each participant.

We designed our dataset to be as comprehensive as possible, permitting deep phenotyping and allowing multiple research questions to be addressed. We tested 217 community dwelling (not university students) healthy adults (mean age 29 years, range 20–41; 109 females, 108 males) with a wide range of cognitive test performance. Our age range was intended to limit the possible effects of aging (see^16^ for a database designed explicitly to examine aging). We first acquired data concerning participants’ subjective views on their imagination, memory and spatial navigation ability using 10 questionnaires. We complemented these with data from 4 real-world tests known to be associated with the hippocampus – scene imagination, autobiographical memory recall, imagination of the future and spatial navigation. Participants also completed 11 laboratory-based memory tests, and 9 tests of general cognitive functioning. In this dataset we sought to build on the strategy research associated with laboratory-based tasks, to examine the cognitive strategies used in naturalistic tests. We include highly detailed characterisations of the cognitive strategies used in the scene imagination, autobiographical memory recall, imagination of the future and spatial navigation tests, as well a number of the other laboratory-based memory tests. For the questionnaires, cognitive tests and the strategies, as well as providing overall scores, we also supply item/trial level data where appropriate.

This level of detail is mirrored in the range of different types of cutting-edge 3 Tesla (3 T) MRI scans that were also acquired. These include multi-parameter mapping to examine tissue microstructure, diffusion-weighted MRI, T2-weighted high-resolution partial volume structural MRI scans (with isotropic voxels), whole brain resting state functional MRI scans and partial volume high resolution resting state functional MRI scans. The data are provided as NifTI files, as we do not wish to make any assumptions about the data processing pipelines that researchers might want to employ. Using the T2-weighted high-resolution partial volume structural scans, 6 distinct subregions of a participant’s hippocampi were manually segmented – DG/CA4, CA2/3, CA1, subiculum, pre/parasubiculum and uncus. The segmentation protocol we employed^58^ is more comprehensive than many in the field, as it separates DG from CA3, separates the pre/parasubiculum from the subiculum, and includes the uncus. It also involves delineating subfields along the entire length of the hippocampus compared to some protocols that are restricted to the hippocampal body.

With this dataset, we have addressed numerous research^47,52,59–64^ and methods questions^65,66^. This has included showing that the ability to imagine scenes may influence how well we recall our past and imagine our future experiences^60^. While we found no relationships between whole hippocampal volume and autobiographical memory recall ability^63^, examination at the level of the subfields revealed a more nuanced picture. The volume of specifically posterior CA2/3 was related to autobiographical memory ability, but only in those with poorer memory recall^52^. Using the DWI data, we found that variations in MR g-ratio, a measure closely related to conduction velocity, of the parahippocampal cingulum bundle were associated with autobiographical memory recall ability. This tract connects the hippocampus with a range of other brain areas. We further identified two particular features of the parahippocampal cingulum bundle that were linked with autobiographical memory recall ability – inner axon diameter and the extent to which neurites are coherently organised. By contrast, no relationships with myelin thickness were evident^62^.

So much more could be gleaned from this dataset, especially given the important gaps it fills by providing real-world cognitive test data, supplying subjective and strategic data, and by including the manually segmented hippocampal subfield masks for more than 200 people. Cognitive and clinical neuroscientists researching individual differences, real-world cognition, brain-behaviour associations, hippocampal subfields, connectivity, to name but a few, will find this dataset ripe for testing their hypotheses with high quality data at scale.

## Methods

In this section we begin by describing the participants, detailing the recruitment and screening procedures and summarising the demographics of the final sample (n = 217). An outline of the testing procedure follows. We then describe the questionnaires that participants completed. This is followed by a description of the cognitive tests that were performed, starting with the real-world tests, followed by laboratory-based memory tests, and then laboratory-based general cognitive tests. Next, we outline how data were collected in order to characterise the strategies used to perform the cognitive tasks. Finally, we describe the MRI data, detailing each type of scan in turn.

### Participants

The data were collected between March 2015 and June 2017. The study was approved by the University College London Research Ethics Committee (project ID: 6743/001) before recruitment began. Individuals expressing an interest in taking part in the study were emailed the study information sheet and were telephoned to assess their initial eligibility. Potentially eligible participants then completed additional screening questions (see Recruitment and screening section below), having provided their consent to do so. Participants recruited into the study proper following eligibility checks were reimbursed £10 per hour for taking part which was paid at study completion. Recruited participants provided written informed consent to take part in each aspect of the study and to share their anonymised data.

The final sample comprised 217 people (for full details see Sample demographics below). A sample size of 217 participants was determined during study design to be robust to employing different statistical approaches when answering multiple questions of interest. For example, the sample allowed for sufficient power to identify medium effect sizes when conducting correlation and regression analyses, and when comparing multiple groups using ANOVAs at alpha levels of 0.01 and when comparing correlations at alpha levels of 0.05^67^. In addition, the sample size was large enough to conduct mediation analyses and structural equation modelling^68^, as well as samples of over 200 participants being suggested as sufficient for correlational neuroimaging research examining individual differences^14,15^.

### Recruitment and screening

This study aimed to recruit individuals from the general population. Consequently, we went to great lengths to recruit people who do not typically take part in scientific research, i.e. the ordinary community dweller. Recruitment involved placing advertisements in the local media (both print and online), flyers and posters located in hundreds of locations across London, UK including (but not limited to) hairdressers, cafes, pubs, convenience stores, local libraries, social clubs, community centres, sports club and laundrettes. Societies and groups based in and around London were contacted via telephone and/or email with requests to send information to members. Work places, including department stores, grocery stores, recruitment agencies and gyms, were approached to disseminate study information to their workers.

Telephone and online screening were used to assess eligibility. Participants were recruited if they were aged 20–41 (to limit the possible effects of aging), had English as their first language, were MRI compatible, had no self-reported psychological, psychiatric or neurological health conditions (e.g. depression, epilepsy), and did not have extreme expertise on classic hippocampal tasks, as this can affect hippocampal structure^5,69^. This later factor excluded individuals with vocations such as taxi driving (or those training to be taxi drivers), ship navigators, aeroplane pilots, or those with regular hobbies including orienteering, or taking part in memory sports and competitions. As a further measure of psychological well-being, potential participants also completed the Beck Depression Inventory Second Edition^70^. This questionnaire assesses current depression levels, with participants responding to 21 questions on a four-point scale asking about their mood over the last two weeks. Inclusion in the study required a score of less than 14 (scores greater than or equal to 14 can be indicative of depressed mood).

### Sample demographics

The final sample of 217 individuals included 109 females and 108 males, with a mean age (at testing) of 29.0 years (*SD* = 5.60). Age and gender were balanced across the sample; 109 participants were aged 20–29 years, of which 54 were male and 55 were female, and 108 participants were aged 30–41 years, 54 being male and 54 being female. Participants were also allocated to one of three magnetic resonance imaging (MRI) scanners (see MRI data section below) located at the same imaging centre, with age and gender again being equally distributed across the scanners. According to self-reported ethnic group information, 72% of the sample identified as White, 7% identified as Black or Black British, 6% identified as Asian, 5% identified as Mixed race and 9% identified as Other. Note that due to concerns regarding re-identification we do not provide ethnicity data at the individual participant level.

The following additional demographic information was collected:

#### Handedness

Participants were asked for their dominant hand, reporting either “right” or “left”. Right hand dominant participants numbered 197 (90.78% of the sample), with 20 being left hand dominant (9.22%). This is in line with worldwide estimates that approximately 10% of people are left handed^71,72^. These proportions were confirmed from data collected using the Edinburgh Handedness Inventory^73^. Note that due to concerns regarding re-identification we do not provide handedness data at the individual participant level.

#### Languages spoken

Information about whether participants could fluently speak other languages (in addition to English) was also obtained. This was scored as either “1” (only English spoken) or “2” (English and other languages spoken). Being monolingual was reported by 179 people (82.49% of the sample), with 38 people (17.51%) speaking at least one additional language fluently. Note that due to concerns regarding re-identification we do not provide languages spoken data at the individual participant level.

#### Years of education

This was measured based on the English education system. Individuals leaving formal education at the earliest permitted time point, at age 16 after completing GCSE examinations, had completed 11 years of full-time education. Those leaving at age 18 after completing A-levels had undergone 13 years of full-time education. Years in any additional courses following this (e.g. degree, apprenticeship) were then added. Part-time qualifications were also included, with each year of part-time education being counted as half a year. Across the sample the average years of education was 16.20 years (SD = 1.99).

### Study procedure

Following recruitment into the study, participants performed an online battery of questionnaires. They then had four in-person visits to our Centre. During visits 1 and 2, participants underwent MRI scanning and performed cognitive tasks. Visit 3 consisted solely of cognitive testing. Visit 4 involved examining the strategies participants used to perform the cognitive tasks. The order of tests within each visit was the same for all participants (see the Test order section). Each visit was approximately the same length, lasting 3–3.5 hours, including breaks. All participants completed all parts of the study. The average time elapsed between visit 1 and visit 2 was 6.93 days (SD = 6.72), between visits 2 and 3 was 5.77 days (SD = 4.43), and between visits 3 and 4 was 6.05 days (SD = 5.33). Between visits 1 and 4, the average length of elapsed time was 15.56 days (SD = 7.77) and between visits 2 and 4 was 8.54 days (SD = 5.92).

### Questionnaires

Participants completed ten questionnaires. We describe each questionnaire, and its subscales, in alphabetical order. For ease of reference, Table 1 also lists the questionnaires and their subscales also in alphabetical order.

**Table 1:** The questionnaires used and their subscales, presented in alphabetical order.

Questionnaire	Subscales
Edinburgh Handedness Inventory	
Memory Experience Questionnaire (MEQ)	MEQ Accessibility
	MEQ Coherence
	MEQ Distancing
	MEQ Emotion
	MEQ Sensory
	MEQ Sharing
	MEQ Time Perspective
	MEQ Visual Perspective
	MEQ Vividness
Object-Spatial Imagery and Verbal Questionnaire (OSIVQ)	OSIVQ Object-Scene
	OSIVQ Spatial
	OSVIQ Verbal
One Sentence Questionnaire	Imagery Ability
	Imagery Use
	Imagery as a Scene
	Memory Ability
	Memory in Imagery
	Memory in Scene Imagery
	Memory in Words
	Future Thinking Ability
	Future Thinking in Imagery
	Future Thinking in Scene Imagery
	Future Thinking in Words
	Navigation Ability
	Navigation in Imagery
	Navigation in Scene Imagery
	Navigation in Words
Plymouth Sensory Imagery Questionnaire (PSIQ)	PSIQ Appearance
	PSIQ Sound
	PSIQ Smell
	PSIQ Taste
	PSIQ Touch
	PSIQ Bodily Sensation
	PSIQ Taste
Santa Barbara Sense of Direction Scale	
Spontaneous Use of Imagery Scale	
Subjective Memory Questionnaire	
Survey of Autobiographical Memory (SAM)	SAM Episodic
	SAM Future
	SAM Spatial
	SAM Semantic
Visualizer –Verbalizer	Visual Items
	Verbal Items
	Dream Items

#### Edinburgh handedness inventory

The Edinburgh Handedness Inventory^73^ assesses the dominance of a person’s right or left hand in everyday activities. Ten activities are provided (e.g. writing, drawing, using scissors) and participants indicate their hand preference on a 4 point scale: 1 (strong preference for left); 2 (preference for left); 3 (preference for right); 4 (strong preference for right). The 10 items are then totalled and converted to an overall score from −100 (strong preference for left) to +100 (strong preference for right).

#### Memory experience questionnaire (MEQ)

The MEQ^74^ assesses the phenomenology of autobiographical memory across different dimensions. The original questionnaire asks participants to focus on a specific past event. For our purposes, this was adapted to concern the recall of autobiographical memories in general.

The questionnaire examines ten dimensions. The subscales for each dimension consist of varying numbers of statements which participants rate on a 5 point scale from 1 (strongly disagree) to 5 (strongly agree). Scores are calculated for each subscale by totalling the responses to each statement within the subscale. High scores represent high self-reported ability. Questions from all subscales are intermixed throughout the questionnaire.

*MEQ accessibility*. This subscale consists of five statements. Two are positively scored (e.g. “Memories are easy for me to recall”) and three are reverse scored (e.g. “It is difficult for me to think of past events”). The total score is out of 25.

*MEQ coherence*. This subscale comprises eight statements. Four are positively scored (e.g. “I recognize the setting in which my memories take place”) and four are reverse scored (e.g. “I have a difficult time remembering events in a coherent manner”). The total score is out of 40.

*MEQ distancing*. This subscale comprises six statements. Three are positively scored (e.g. “I don’t have much in common with the person in my memories.”) and three are reverse scored (e.g. “My memories are consistent with who I think I am today.”). The total score is out of 30.

*MEQ emotion*. This subscale consists of six statements. Three are positively scored (e.g. “My memories of events evoke powerful emotions.”) and three are reverse scored (e.g. “I do not have strong emotions about my personal memories.”). The total score is out of 30.

*MEQ sensory*. This subscale consists of eight statements. Four items are positively scored (e.g. “As I remember events, I can hear them in my mind.”), and four are reverse scored (e.g. “My memories do not involve a lot of sensory information (sounds, smells, tastes, etc.).”). The total score is out of 40.

*MEQ sharing*. This subscale consists of six statements. Three are positively scored (e.g. “I frequently think about or talk about past events with others”) and three are reverse scored (e.g. “I rarely tell others about my memories”). The total score is out of 30.

*MEQ time perspective*. This subscale comprises six statements. Three are positively scored (e.g. “My memory for the hour when events took place is clear”) and three are reverse scored (e.g. “My memory for the day when events took place is vague”). The total score is out of 30.

*MEQ valence*. This subscale comprises six statements. Three are positively scored (e.g. “My past experiences have been positive”) and three are reverse scored (e.g. “The experiences described in my memories are negative.”). The total score is out of 30.

*MEQ visual perspective*. This subscale consists of six statements. Three are positively scored (e.g. “In my memories, I see experiences through my own eyes”) and three are reverse scored (e.g. “I experience memories as if I was an observer to the event”). The total score is out of 30.

*MEQ vividness*. This subscale consists of six statements. Three items are positively scored (e.g. “My memory for events is very vivid”), and three are reverse scored (e.g. “My memory for events is dim”). The total score is out of 30.

#### Object-spatial imagery and verbal questionnaire (OSIVQ)

The OSIVQ^75^ is designed to distinguish between different types of imagery users and has three subscales, two related to visual imagery and one to verbal processing. The Object subscale measures the ability to imagine vivid and detailed images of objects and scenes. The Spatial subscale measures the ability to process locations, movement and transformations, often represented by more technical and schematic imagery. The Verbal subscale measures the use of verbal strategies.

For our study, we renamed the Object subscale “Object-Scene” in order to better represent what the scale is designed to measure. Object imagery typically suggests an image of an object devoid of any background. However, as stated by the authors^75^, the Object subscale is not limited to individual objects but can also refer to imagery of patterns and scenes, characterising their colour, vividness, shape and details.

Each of the three subscales of the OSIVQ contains 15 statements. The participant is asked to rate each statement on a five point scale from 1 (totally disagree) to 5 (totally agree). The final score on each subscale is the average of the responses over the 15 items. High scores represent high self-reported ability.

*OSIVQ object-scene*. For this subscale, statements include: “When reading fiction, I usually form a clear and detailed mental picture of a scene or room that has been described” and “I can close my eyes and easily picture a scene that I have experienced”. No statements are reverse scored.

*OSIVQ spatial*. For this subscale, statements include: “My images are more like schematic representations for things and events rather than detailed pictures” and “I can easily sketch a blueprint for a building that I am familiar with”. One statement in the subscale is reverse scored (“I find it difficult to imagine how a three-dimensional geometric figure would exactly look like when rotated”).

*OSVIQ verbal*. For this subscale, statements include: “When remembering a scene, I use verbal description rather than mental pictures” and “I am always aware of sentence structure”. Three statements in the subscale are reverse scored (e.g. “I have difficulty expressing myself in writing”).

#### One sentence questionnaire

The one sentence questionnaire was developed and tested using the participants in the current cohort^61^. The questionnaire aimed to gain a broad profile of a person in a short time frame. It consists of 15 questions covering four areas of cognition – imagination, autobiographical memory, future thinking and navigation. The questions are as follows:

*Imagery ability*. “Please rate your ability to construct a mental image”. Answers are on a 7 point scale from 1 (very high) to 7 (very low). This is reverse scored so that a high ability is a high score.

*Imagery use*. “In everyday life, how much do you think in images (e.g. thinking in pictures in your mind)?” Answers are on a 7 point scale from 1 (not at all) to 7 (all the time).

*Imagery as a scene*. “If you think in images, to what extent does this involve spatially coherent scenes (e.g. scenes that you could step into or operate within) compared to single objects?” Answers are on a 7 point scale from 1 (single objects) to 7 (coherent scenes).

*Memory ability*. “Please rate your ability to remember your personal past”. Answers are on a 7 point scale from 1 (very high) to 7 (very low). This is reverse scored so that a high ability is a high score.

*Memory in imagery*. “When recalling the past, to what extent do you think in images?” Answers are on a 7 point scale from 1 (not at all) to 7 (all the time).

*Memory in scene imagery*. “If you think in images when recalling the past, to what extent do you evoke spatially coherent scenes in your mind’s eye, compared to imagining single objects?” Answers are on a 7 point scale from 1 (single objects) to 7 (coherent scenes).

*Memory in words*. “When recalling the past, how much do you think verbally (e.g. thinking in words and sentences)?” Answers are on a 7 point scale from 1 (not at all) to 7 (all the time).

*Future thinking ability*. “Please rate your ability to imagine future events”. Answers are on a 7 point scale from 1 (very high) to 7 (very low). This is reverse scored so that a high ability is a high score.

*Future thinking in imagery*. “When imagining the future, to what extent do you think in images?” Answers are on a 7 point scale from 1 (not at all) to 7 (all the time).

*Future thinking in scene imagery*. “If you think in images when imagining the future, to what extent do you evoke spatially coherent scenes in your mind’s eye, compared to imagining single objects?” Answers are on a 7 point scale from 1 (single objects) to 7 (coherent scenes).

*Future thinking in words*. “When imagining the future, how much do you think verbally (e.g. thinking in words and sentences)?” Answers are on a 7 point scale from 1 (not at all) to 7 (all the time).

*Navigation ability*. “Please rate your navigational ability”. Answers are on a 7 point scale from 1 (very high) to 7 (very low). This is reverse scored so that a high ability is a high score.

*Navigation in imagery*. “When you navigate, to what extent do you think in images?” Answers are on a 7 point scale from 1 (not at all) to 7 (all the time).

*Navigation in scene imagery*. “If you think in images when navigating, to what extent do you evoke spatially coherent scenes in your mind’s eye, compared to imagining single objects?” Answers are on a 7 point scale from 1 (single objects) to 7 (coherent scenes).

*Navigation in words*. “When navigating, how much do you think verbally (e.g. thinking in words and sentences)?” Answers are on a 7 point scale from 1 (not at all) to 7 (all the time).

#### Plymouth sensory imagery questionnaire (PSIQ)

The PSIQ^76^ measures imagery ability across seven sensory modalities. Each subscale requires participants to imagine three scenarios. They then rate the sensory image generated on an 11 point scale from 0 (no image at all) to 10 (vivid as real life). Scores on the three scenarios are summed to create a total score out of 30 for each subscale. An overall score out of 210 can also be calculated by summing the scores from all of the subscales. High scores reflect high self-reported ability.

*PSIQ appearance*. Participants are asked to imagine the appearance of a bonfire, a sunset, and a cat climbing a tree.

*PSIQ sound*. Participants are asked to imagine the sound of a car horn, applause, and an ambulance siren.

*PSIQ smell*. Participants are asked to imagine the smell of newly cut grass, burning wood, and the smell of a rose.

*PSIQ taste*. Participants are asked to imagine the taste of black pepper, lemon, and mustard.

*PSIQ touch*. Participants are asked to imagine touching fur, warm sand, and a soft towel.

*PSIQ bodily sensation*. Participants are asked to imagine the bodily sensation of relaxing in a warm bath, walking briskly in the cold, and jumping into a swimming pool.

*PSIQ taste*. Participants are asked to imagine feeling excited, relieved, and scared.

#### Santa barbara sense of direction scale

The Santa Barbara Sense of Direction Scale^77^ assesses spatial and navigational abilities, preferences and experiences. Fifteen statements are presented, with participants indicating their level of agreement with each statement. Ratings are made on a 7 point scale from 1 (strongly agree) to 7 (strongly disagree). Seven statements are positively coded (e.g. “I am very good at giving directions”) and eight are reverse scored (e.g. “I don’t have a very good “mental map” of my environment”). Scores are summed across the 15 statements, with a low score indicating good navigation ability, and a high score reflecting poor navigation ability.

#### Spontaneous use of imagery scale (SUIS)

The SUIS^78^ consists of 12 statements that measure how frequently an individual uses visual imagery. Participants read each statement and indicate the degree to which the statement is appropriate to them. Each statement is rated on a 5 point scale from 1 (never) to 5 (always). Example statements include: “If I am looking for new furniture in a store, I always visualize what the furniture would look like in particular places in my home” and “When I hear a radio announcer or DJ I’ve never actually seen, I usually find myself picturing what they might look like.” Scores are summed across the 12 items to give a final score out of 60. High scores reflect high self-reported ability.

#### Subjective memory questionnaire

The Subjective Memory Questionnaire^79^ probes memory for things people often try to remember. It is split into two sections. First, is a list of 36 items (e.g. “telephone numbers”; “jokes”; “birthdays”) which participants rate in response to the question “How good is your memory for…?” Answers are given on a 5 point scale from 1 (very bad) to 5 (very good). The second section asks the question “How often do you…?” in relation to seven experiences (e.g. “Set off to do something, then can’t remember what”; “Forget whether or not you have locked up the house”). Answers are provided on a 5 point scale from 1 (very rarely) to 5 (often), and are reversed scored. The total score is the sum of all responses (out of 215). High scores reflect high self-reported ability.

#### Survey of autobiographical memory (SAM)

The SAM^80^ assesses episodic and semantic aspects of autobiographical memory, as well as future thinking and spatial memory. There are 26 items in total. Participants respond on a five point scale regarding the extent to which they agree with each statement, from 1 (completely disagree) to 5 (completely agree). Scoring is determined via a weighting system. Responses are weighted and calculated together to provide an average score that centres around 100, like an IQ, with high scores reflecting high self-reported ability. Full details of the weighting and scoring procedure are available from the SAM authors.

*SAM episodic*. This subscale assesses autobiographical memory recall. It contains 8 statements (e.g. “I am highly confident in my ability to remember past events”), two of which are reversed scored (e.g. “Specific events are difficult for me to recall”).

*SAM future*. This subscale examines a participant’s ability to imagine future events. It contains 6 statements (e.g. “When I imagine an event in the future, the event generates vivid mental images that are specific in time and place”), one of which is reverse scored (e.g. “I have a difficult time imagining specific events in the future”).

*SAM spatial*. This subscale assesses navigation ability. It contains 6 statements (e.g. “In general, my ability to navigate is better than most of my family/friends”), two of which are reverse scored (e.g. “I get lost easily, even in familiar areas”).

*SAM semantic*. This subscale probes the ability to recall facts and information. It contains 6 statements (e.g. “I can learn and repeat facts easily, even if I don’t remember where I learned them”), two of which are reverse scored (e.g. “I have a hard time remembering information I have learned at school or work”).

#### Visualizer –verbalizer

This questionnaire^81^ assesses an individual’s preference for visual or verbal learning styles, with a third subscale focused on dream imagery. Thirty statements are provided, 10 corresponding to visual items, 10 to verbal items and 10 to dream items. The participant indicates for each item whether, for them, the statement is true or false. Half of the statements are phrased positively, in that an answer of “true” reflects a visual or verbal learning preference or vivid dream imagery (e.g. visual learning style: “The old saying ‘A picture is worth a thousand words’ is certainly true for me”; verbal learning style: “I have better than average fluency in using words”; dream imagery: “My dreams are extremely vivid”). The other half are phrased negatively where an answer of “false” reflects a visual or verbal learning preference or vivid dream imagery (e.g. visual learning style: “I seldom use diagrams to explain things”; verbal learning style: “I dislike word games like crossword puzzles”; dream imagery: “I seldom dream”). The three scales are scored separately. The final score (out of 10 for each scale) is the number of responses reflecting the stated topic, with high scores reflecting high self-reported ability.

#### Order of questionnaires

The questionnaires were presented to participants in the following order: Edinburgh Handedness Inventory, One Sentence Questionnaire, Santa Barbara Sense of Direction Scale, Spontaneous Use of Imagery Scale, SMQ, Visualizer–Verbalizer, OSIVQ, SAM, PSIQ, MEQ.

### Real-world tests

Participants were tested using four real-world tasks assessing imagination, autobiographical memory recall, future thinking and navigation. A list of the specific tests is shown in Table 2. A full list of the sub-measures of each test is provided in Supplementary Table 1.

**Table 2:** The real-world tests.

Real-world Tests
Scene Construction Test
Autobiographical Interview
Future Thinking Test
Navigation Test

#### Scene construction test

The Scene Construction Test^11^ measures a participant’s ability to mentally construct an atemporal visual scene, meaning that the scene is not grounded in the past or the future. Participants construct seven different scenes of commonplace settings (the swimming pool of a luxury hotel; a busy fishing harbour; an old library; the boardroom of a big corporation; the ruins of a derelict building; a large circus tent; the inside of an ancient cathedral). For each scene, a short cue is provided (e.g. “imagine you’re lying by the side of a swimming pool of a luxury hotel”), and the participant is asked to imagine the scene that is evoked and then describe it out loud in as much detail as possible. Participants are explicitly told not to describe a memory, but to create a new scene that they have never experienced before. Participants give descriptions until they come to a natural end or cannot add any additional details. If required, a probing protocol is utilised to attempt to elicit more details (if a description is particularly poor). These are either very general probes (“is there anything else you can tell me?”) or based upon a theme described by the participant. The experimenter is never allowed to introduce new concepts or details that have not been mentioned by the participant. All descriptions are audio recorded and transcribed for scoring.

*Experiential Index*. The overall outcome measure of this test is the Experiential Index, a composite measure of the overall richness of the imagined scenario. The Experiential Index is composed of four elements (described in detailed below): the content, participant ratings of their sense of presence (how much they felt like they were really there) and perceived vividness, participant ratings of the spatial coherence of the scene, and an experimenter rating of overall quality of the scene. Experiential Index scores range from 0 (not experienced at all) to 60 (extremely richly experienced), with 28 points from the content, 8 points from participant ratings, 6 points from the spatial coherence and 18 points from the quality rating. An Experiential Index score is calculated for each scene and then averaged to provide a single final outcome measure. High scores represent high scene construction ability.

*Content*. To obtain the content score, the transcribed descriptions of each scene are split into statements. These are then classified by the experimenter as belonging to one of four categories (see below). For the Experiential Index, a maximum of seven details per category is allowed (providing a maximum content score of 28). The original study reporting the development and use of this test determined that seven details per category was sufficient to create a coherent scene without over-rewarding more verbose participants^11^.

Content: spatial references. The spatial references category refers to statements regarding the relative position of entities within the environment, directions relative to a participant’s vantage point, or explicit measurements (“behind the bar” or “to my left I can see” or “the ceiling is about 40 feet high”).

Content: entities present. The entity category is a simple count of how many distinct entities (e.g. objects, people, animals) were mentioned (“I can see some birds”).

Content: sensory descriptions. The sensory descriptions category consists of any statements describing (in any modality) properties of an entity (“the chair I’m sitting on is made of wood”) as well as general weather and atmosphere descriptions (“it is very hot” or “the room is very smoky”).

Content: thought/emotions/actions. The thought/emotion/action category concerns any introspective thoughts or emotional feelings (“I have a sense of being alone”) as well as the thoughts, intentions, and actions of other entities in the scene (“he seems to be in a hurry”).

*Participant Ratings included in the experiential index*. Participants also complete two self-report ratings regarding each imagined scene that are included in the experiential index.

Participant rating: sense of presence. Participants first rate the imagined scene in terms of their feeling of sense of presence. They do so on a 5 point scale in response to the question “How much of a sense of being there did you have when imagining?” from 1 (I did not feel like I was there at all) to 5 (I felt strongly like I was really there). For the Experiential Index, this is rescaled to 0–4.

Participant rating: vividness. Participants also rate the vividness of their imagined scenario. This is done on a five point scale in response to the question “How vivid was the scene you imagined in your mind’s eye?” from 1 (I couldn’t really see anything) to 5 (extremely vivid). For the Experiential Index, this is rescaled to 0–4.

*Spatial coherence*. The spatial coherence metric measures the extent to which the patients felt like the imagined experiences were taking place in an integrated and coherent spatial context as opposed to merely being a fragmented collection of images. After each scenario, participants are presented with twelve statements, each providing a possible qualitative description of the imagined experience. Participants are instructed to indicate which statements they feel accurately describe their construction. They are free to identify as many or as few as they think is appropriate.

Spatial coherence raw. To calculate the spatial coherence raw score, one point is awarded for each coherent statement selected (e.g. “I could see the whole scene in my mind’s eye”) and one point taken away for each fragmented statement (e.g. “It was a collection of separate images”), yielding a score between –4 and + 8. A high score reflects a coherent scene.

Spatial coherence normed. A spatial coherence normed score is then calculated by normalising the raw score around zero, providing a value ranging between –6 and + 6.

Spatial coherence index. For inclusion in the Experiential Index, a score consisting of only positive spatial coherence normed values is utilized, referred to as the Spatial Coherence Index. Only positive values are used so as not to over penalise fragmented descriptions.

*Experimenter rating: quality*. The experimenter also assesses the overall quality of the imagined construction. This is rated on a 11 point scale in response to how much the experimenter feels the description evokes a detailed picture of the experience in their own mind’s eye, ratings ranging from 0 (the construction was completely devoid of details and with no sense of experiencing) to 10 (an extremely rich and highly evocative construction that appeared to emerge from an extremely vivid imagining). For use in the Experiential Index this is rescaled to 0 to 18.

*Additional participant ratings*. Three additional participants ratings are also collected for each scene. These are not included in the Experiential Index.

Participant rating: difficulty. The first rating asks how difficult participants found imaging the scene, responding to the question “How difficult did you find this task?” on a 5 point scale from 1 (very easy) to 5 (very hard).

Participant rating: detail. The second rating concerns how detailed participants thought their scene was, responding to the question “How detailed do you feel your description of the scene was?” on a 5 point scale from 1 (hardly any details at all) to 5 (extremely richly detailed).

Participant rating: memory similarity. Finally, participants rate how similar the imagined scenario was to a real memory, responding to the question “How similar to memories that you can recall was your imagined scene?” on a 5 point scale from 1 (exactly like a memory) to 5 (nothing at all like any memories I can recall).

#### Autobiographical interview

The widely used Autobiographical Interview^82^ was employed to measure autobiographical memory recall ability.

Participants are asked to provide autobiographical memories from a specific time and place over four time periods – early childhood (up to age 11), teenage years (aged from 11–17), adulthood (age from 18 to 12 months prior to the interview; two memories were requested) and the last year (a memory in the last 12 months). In our study, participants were asked to avoid selecting a memory from the last 12 months in the adulthood category to ensure that only the last year category contained memories from the last 12 months.

Participants are asked to select events that they are comfortable to talk about. They are told that the event has to be one they were personally involved in and one that they could recollect (they could not just have been told about the event by others). All memories have to be from a specific time and place – an event, for example, could not be a two week summer holiday, but a specific event on that holiday would be acceptable. Participants are first asked to simply describe and speak about the event selected. This occurs without interruption from the experimenter until they have reached a natural end point. On completion, the experimenter prompts the participant with a general probe (e.g. “Is there anything more you can tell me?”) to see if any additional details can be elicited. All memories are audio recorded and transcribed for analysis.

For scoring, each memory is divided into segments of information. Segments are defined as a specific occurrence, observation or thought. Two main groups of details are identified – Internal (episodic) details or External (non-episodic) details. Internal details are those describing the event in question, are specific to a time and place, and are considered to reflect episodic re-experiencing. External details describe semantic information concerning the event or non-event information. Within each overarching Internal details and External details category are subcategories (five for Internal details and four for External details). In addition, a series of ratings are also performed for each memory; six by the experimenter and five by the participant. Each measure is scored for each memory and then averaged across the five memories collected to provide an overall metric. The available scores are therefore as follows:

*Internal total*. Internal total is the sum of all Internal details for the memory recalled.

*Internal events*. Internal events are happenings central to the story, including individuals present, weather conditions, physical/emotional actions, or reactions.

*Internal time*. This category includes all information relating to when the event occurred, for example, the year, season, month, day of week, time of day.

*Internal place*. Internal place is the location of the memory, including the city, street, building, room or part of a room.

*Internal perceptual*. Internal perceptual covers all sensory information; auditory, olfactory, tactile, taste, and visual details, body positions (e.g. sitting/standing) and duration.

*Internal emotion*. This final category concerns emotional states, thoughts and implications.

*External total*. This is the sum of all External details for the memory recalled.

*External event*. External events are specific details from other memories, and that are not describing the main event recalled.

*External semantic*. These are segments detailing general knowledge or facts, ongoing events or extended states of being.

*External repetition*. External repetitions are any unsolicited repetition of details (from any Internal or External category).

*External other*. This final category relates to metacognitive statements and editorializing.

*Experimenter ratings*. Six experimenter ratings are collected for each memory. With the exception of episodic richness, these are scored on 4 point scales from 0 (no mention of information pertaining to the specified category) to 3 (a rich, highly specific, evocative, and/or vivid description that appears to emerge from a feeling of re-experiencing).

Experimenter rating: episodic richness. The episodic richness rating is the overall degree to which a feeling of re-experiencing was evoked. This rating is performed on a 7 point scale from 0 (no mention of information pertaining to the specified category) to 6 (a rich, highly specific, evocative, and/or vivid description that appears to emerge from a feeling of re-experiencing) to provide a finer grained rating and to account for the greater importance of this category relative to the others.

Experimenter rating: time. This rating concerns information relating to when the event occurred, including the year, season, month, day of week, time of day.

Experimenter rating: place. This rating relates to information associated with the location of the event, including the city, street, building, room or part of a room.

Experimenter rating: perceptual. This rating concerns the extent of sensory information provided including auditory, olfactory, tactile, taste, and visual details.

Experimenter rating: emotion. This rating relates to emotional states, thoughts and implications.

Experimenter rating: time integration. The time integration rating aims to capture the extent to which a participant integrates the recalled episodic event into a larger time scale, for example, by providing temporal contextual information or relating it to other life periods.

*Participant ratings*. After recalling their memories, participants are also asked to answer five questions concerning their recall.

Participant rating: how clearly visualize. This rating measures how well the participants can visualise their autobiographical memory, responding to the question “How clearly can you visualize this event?” on a 6 point scale from 1 (vague memory, no recollection) to 6 (extremely clear, as if it was happening now).

Participant rating: emotional change during event. This rating focuses on changes in the participants’ emotional state during the event, with participants responding to the question “How much did your emotional state change from before the event occurred to after it happened?” on a 6 point scale from 1 (no change in how I felt) to 6 (underwent tremendous emotional change).

Participant rating: importance of event now. This rating measures the importance of the event to the participant at the current time. Participants answer the question “How personally important is this event to you now?” on a 6 point scale from 1 (no importance at all) to 6 (of great importance).

Participant rating: importance of event then. Participants are then asked about the importance of the event to them at the time it occurred. Participants answer the question “How personally important was this event to you then?” on a 6 point scale from 1 (no importance at all) to 6 (of great importance).

Participant rating: how often think about the event. Finally, participants are asked about how often they rehearse the recalled event, responding to the question “On average, how often do you think or talk about this event?” selecting from one of six categorical answers: 1 (once every few years); 2 (once per year); 3 (every 6 months); 4 (every 3 months); 5 (every month); 6 (once per week).

#### Future thinking test

This test^11^ follows the same procedure as the Scene Construction Test but requires participants to imagine three plausible future scenes involving themselves (an event at the weekend; next Christmas; the next time they will meet a friend). Unlike scenes in the Scene Construction Test, scenes in the future thinking task involve “mental time travel” to the future, so they have a clear temporal dimension. Participants are explicitly told not to describe a memory, but to create a new future scene. Recordings are transcribed for later scoring. The scoring procedures are the same as for the Scene Construction Test.

#### Navigation test

Navigation ability was assessed using movies of navigation through an unfamiliar town^83^. Movie clips of two overlapping routes through this real town (Blackrock, in Dublin, Ireland) are shown to a participant four times. Footage is unique to each route apart from one crossover point at a large road junction. The footage was shot at eye level and proceeds at an average walking pace, with the camera panning from side to side to simulate viewing and to pick up the features and landmarks along the routes. At road junctions, the pace is slowed to allow for all elements of the junction to be observed before continuing. The movies are shown without sound. Participants are told to focus on salient landmarks to help them learn the town, ignoring cars, buses and people. Landmarks are defined as prominent buildings and distinctive elements of the route. Participants are explicitly told that the two routes, while shown separately, overlap. Five tasks are used to assess the participant’s ability to learn this town.

*Overall navigation score*. An overall navigation score is calculated by combining the scores from all the tasks.

*Clip recognition*. Following each viewing of the route movies, participants are shown four short clips – two from the actual routes, and two distractors. Participants indicate whether they had seen that clip or not. The final score (/16) is the number of correctly identified clips.

*Scene recognition*. After all four route viewings are completed, recognition memory for scenes from the routes is tested. Participants are shown 32 photographs, 24 from the routes (12 from each route) and 8 similar distractors, randomly intermixed. Participants have to report whether they had seen that scene or not. The final score (/32) is the number of correctly identified scenes.

*Proximity judgements*. The third test involves assessing knowledge of the spatial relationships between landmarks from the routes. On each trial, three colour photographs of landmarks are presented and participants have to judge which of two of the landmarks was closer, as the crow flies, to the third picture (i.e. the target landmark). Ten trials are conducted, 6 where the landmarks are all from the same route (3 from each route) and 4 where the landmarks are from across the two routes. The final score is the number of correct judgements (/10).

*Route knowledge*. In this test, route knowledge is examined by having participants place photographs from the routes in the correct order as if travelling through the town. On each trial, participants are given eight photographs, one marked as the “start point” and another as the “end point”. They are then asked to place the other six photographs in the correct order to get from the start to the end. Four trials are performed, two that remain within one route and two that involve both routes. Correctly placed photographs are given a score of 1 (the maximum being 24).

*Sketch map*. Finally, participants draw a sketch map of the two routes including as many landmarks as they can remember (with it being made clear that drawing ability was not being assessed). Sketch maps are scored in terms of:

Number of road segments. This is a count of the number of road segments on the sketch map, a segment being the section of road between road junctions, with a maximum score of 16.

Number of road junctions. This is a count of the number of road junctions correctly included. Road junctions being where side roads branch from the two main roads, and the main junction where the two roads of interest overlap. Eight road junctions are present.

Number of landmarks. Within the two routes 34 identifiable landmarks are present. The number of correctly included landmarks (regardless of location) is counted.

Landmark placement. This score focuses on the correct location of the landmarks. Three points were available for each landmark; 1 for the correct side of the road, 1 for placement with regards to nearby road junctions and 1 for being in the correct sequence of nearby landmarks, providing a total maximum score of 102.

Orientation rating. This is an experimenter rating assessing the orientation and layout of the map on a 5 point scale from 1 (a poor representation of the town) to 5 (an accurate orientation of the town).

Overall categorization. This is an experimenter score representing map coherence. The experimenter chose from one of six possibilities: 1 (the two routes were merged); 2 (two routes were present, but drawn separately); 3 (routes were close together but not joined accurately); 4 (some elements of the routes were linked, but integration was mainly lacking); 5 (the two routes were integrated, but some inaccuracies); 6 (correct integration, easy to follow and use for navigating).

### Laboratory-based memory tests

Participants performed 11 laboratory-based memory tests. A list of the specific tests is shown in Table 3. A full list of the sub-measures for each test is provided in Supplementary Table 2.

**Table 3:** The laboratory-based memory tests.

Laboratory-Based Memory Tests
Rey–Osterrieth Complex Figure Test
Object–Place Association Test
Rey Auditory Verbal Learning Test
Logical Memory Test
Wechsler Memory Scale Verbal Paired Associates Test
Concrete Verbal Paired Associates Test
Abstract Verbal Paired Associates Test
Warrington Recognition Memory Tests for Words Warrington Recognition Memory Tests for Faces Warrington Recognition Memory Tests for Scenes Dead or Alive Test

#### Rey–osterrieth complex figure test

This test^84^ assesses visuospatial processing and memory. Participants are first asked to copy the figure (a complicated line drawing with multiple components), reproducing the figure freehand. Thirty minutes later, participants are asked to draw the same figure from memory. Participants are not told in advance that they will have to reproduce the figure at a later point. Scores are determined via the presence and placement of the 18 components in the figure, with 2 points available for each component, providing a maximum score of 36. Two outcome measures are provided.

*Copy score*. The total score when a participant copies the figure, maximum score of 36.

*Delay recall score*. The total score when a participant draws the figure from memory, maximum score of 36.

#### Object–place association test

This test was adapted for the computer based on a previously used Object–Place Association Test^85^. Participants are presented with 16 coloured objects on a white background with black edging. They are initially given 60 seconds to study the location of the objects, following which the objects are removed and just the black edging remains. Participants then have to drag and drop the objects into the correct position, with no limitations on how many times they could move the objects. On completion (indicated by the participant), the original array is re-presented to the participant for another 30 seconds. This is repeated until the participant has seen and attempted to reproduce the array six times. No feedback is given during the test phase. Participants are also asked to reproduce the array after a 30 minute delay. Participants are not told about the delayed recall in advance. Following piloting, an object is deemed to be in the correct location if the centre of the object is placed within 80 pixels (3.2 cm) of the correct centre point. Eight outcome measures are provided for this task.

*Trials to criterion*. This was the number of trials it took a participant to learn the correct position of all the objects. If a participant never learned the location of all 16 objects, a highest score of 7 was awarded.

*Trial 1 score*. The number of correctly located objects on their first attempt to reproduce the array, maximum score of 16.

*Trial 2 score*. The number of correctly located objects on their second attempt to reproduce the array, maximum score of 16.

*Trial 3 score*. The number of correctly located objects on their third attempt to reproduce the array, maximum score of 16.

*Trial 4 score*. The number of correctly located objects on their fourth attempt to reproduce the array, maximum score of 16.

*Trial 5 score*. The number of correctly located objects on their fifth attempt to reproduce the array, maximum score of 16.

*Trial 6 score*. The number of correctly located objects on their sixth attempt to reproduce the array, maximum score of 16.

*Delayed recall score*. The number of correctly located objects when attempting to reproduce the array after a 30 minute delay, maximum score of 16.

#### Rey auditory verbal learning test

This test [see^86^] assesses verbal memory via list learning. Participants hear a list of 15 words and are asked to try and remember as many as possible. The list is read out five times and memory is tested following each reading. After the five repetitions, a different list of 15 words is read out (List B), memory for which is then tested. After this, the participant is asked once again to recall as many words from the original list as possible. Delayed recall of the original list is tested 30 minutes later. Participants are not told about the delayed recall in advance. Nine outcome measures are provided for this task.

*Trial 1 score*. The number of correctly recalled words after hearing the list for the first time, maximum score of 15.

*Trial 2 score*. The number of correctly recalled words after hearing the list for the second time, maximum score of 15.

*Trial 3 score*. The number of correctly recalled words after hearing the list for the third time, maximum score of 15.

*Trial 4 score*. The number of correctly recalled words after hearing the list for the fourth time, maximum score of 15.

*Trial 5 score*. The number of correctly recalled words after hearing the list for the fifth time, maximum score of 15.

*Total immediate recall*. The sum of correctly recalled words from across all five recall trials, maximum score of 75.

*List B recall score*. The number of correctly recalled words from List B, maximum score of 15.

*Interference recall score*. The number of correctly recalled words from the original list when recalled directly after hearing and recalling List B, maximum score of 15.

*Delayed recall score*. The number of correctly recalled words from the original list, recalled after a 30 minute delay, maximum score of 15.

#### Logical memory test

The test is taken from the Wechsler Memory Scale (WMS) IV^87^ and assesses the free recall of narratives. Two short stories are read out to the participant, and the participant is asked to re-tell each story immediately after hearing it. Following a 30 minute delay the participants are asked to recall each story again. Participants are not told about the delayed recall in advance. Points are awarded for each correct piece of information provided. Four outcome measures are available.

*Immediate recall raw score*. The number of correct pieces of information provided from the two narratives when recalled immediately. The maximum score is 50.

*Immediate recall scaled score*. The immediate recall scaled score is calculated from the immediate recall raw score and the age of the participant. The maximum score is 19.

*Delayed recall raw score*. The number of correct pieces of information provided from the two narratives when recalled after a 30 minute delay. The maximum score is 50.

*Delayed recall scaled score*. The delayed recall scaled score is calculated from the delayed recall raw score and the age of the participant. The maximum score is 19.

#### WMS verbal paired associates test

This test is taken from the WMS-IV^87^ and assesses verbal memory for word pairs. Learning takes place over four trials. In each trial, the same 14 word pairs (in a different order each time) are read out to a participant. Following this, the first word of each pair is given and a participant is asked for the corresponding word, with feedback (the correct answer if necessary) provided. After 30 minutes, a participant is tested again in the same way but without feedback. Participants are not told about the delayed recall in advance. Eight outcome measures are available.

*Recall 1*. The number of correctly recalled word pairs after hearing the word pairs for the first time, maximum score of 14.

*Recall 2*. The number of correctly recalled word pairs after hearing the word pairs for the second time, maximum score of 14.

*Recall 3*. The number of correctly recalled word pairs after hearing the word pairs for the third time, maximum score of 14.

*Recall 4*. The number of correctly recalled word pairs after hearing the word pairs for the fourth time, maximum score of 14.

*Total immediate recall raw*. The sum of correctly recalled word pairs from across all four recall trials, maximum score of 56.

*Immediate recall scaled*. The immediate recall scaled score is calculated from the total immediate recall raw score and the age of the participant. The maximum score is 19.

*Delayed recall raw*. The number of correctly recalled word pairs when recalled after a 30 minute delay. The maximum score is 14.

*Delayed recall scaled*. The delayed recall scaled score is calculated from the delayed recall raw score and the age of the participant. The maximum score is 15.

#### Concrete and abstract verbal paired associates tests

The procedures for these two tests are identical, so we outline them both here in one section. We have previously suggested that a limitation of the WMS verbal paired associate task is its reliance upon concrete, imageable words^3,7^. We therefore created two additional versions of this task^60^. In one case, only concrete, imageable words are used while the other comprises only abstract, non-imageable words. The words in each list are highly matched in terms of linguistic characteristics (e.g. length, phonemes and syllables) and frequency use in the English language. Otherwise, the tasks are identical to the WMS verbal paired associates. Six outcome measures are available for each of the Concrete and Abstract verbal paired associates – these are the same as the WMS verbal paired associates but without the scaled scores.

*Recall 1*. The number of correctly recalled word pairs after hearing the word pairs for the first time, maximum score of 14.

*Recall 2*. The number of correctly recalled word pairs after hearing the word pairs for the second time, maximum score of 14.

*Recall 3*. The number of correctly recalled word pairs after hearing the word pairs for the third time, maximum score of 14.

*Recall 4*. The number of correctly recalled word pairs after hearing the word pairs for the fourth time, maximum score of 14.

*Total immediate recall raw*. The sum of correctly recalled word pairs from across all four recall trials, maximum score of 56.

*Delayed recall raw*. The number of correctly recalled word pairs when recalled after a 30 minute delay. The maximum score is 14.

#### Warrington recognition memory tests

The procedures for these three recognition memory tests, involving either words^88^, faces^88^ or scenes^89^, are identical, so we outline them here in one section. In each test 50 stimuli (either words, faces or scenes) are displayed one at a time for 3 seconds. For each item, the participant is asked to judge whether the stimulus is pleasant or unpleasant. A participant is then presented with 50 pairs of stimuli, one of which they saw before and one novel item, and they are asked to indicate which item they saw previously. All stimuli were presented in black and white. Five outcome measures are available.

*Words raw*. The total number of words correctly indicated as being presented previously. Maximum score of 50.

*Words scaled*. The correct number of recognition responses (Words Raw) was converted to a scaled score depending on the age of a participant.

*Faces raw*. The total number of faces correctly identified as being presented previously. Maximum score of 50.

*Faces scaled*. The correct number of recognition responses (Faces Raw) was converted to a scaled score depending on the age of the participant.

*Scenes raw*. The total number of scenes correctly indicated as being presented previously. Maximum score of 50. Note that for the scenes task, no scaled scores exist.

#### Dead or alive test

The Dead or Alive Test^90^ is a test of semantic knowledge. A participant is presented with a list of names of 74 famous individuals and is first asked to remove any names that they do not recognise. For those that a participant knows, they are then asked to indicate whether the individual is dead or alive. As the study was conducted over a two year period, the correct response (dead or alive) had to be updated for some of the individuals included on the list. Three outcome measures are available.

*Number know*. This is the number of individuals on the list that a participant indicates they are familiar with, with the maximum score of 74.

*Number correct*. This is the number of individuals that a participant correctly identifies as being dead or alive. The maximum possible score, if a participant knows all of the individuals on the list, is 74.

*Proportion Correct*. This is the proportion of correct responses given the number of famous individuals known to the participant (i.e. (Number Correct/Number Know) * 100).

### Laboratory-based general cognitive tests

Participants performed nine laboratory-based general cognitive tests. The specific tests are shown in Table 4. A full list of the sub-measures for each test is provided in Supplementary Table 3.

**Table 4:** The laboratory-based general cognitive tests.

Laboratory-Based General Cognitive Tests
Test of Premorbid Functioning
Matrix Reasoning Test
Brixton Spatial Anticipation Test
F-A-S Test
Digit Span Test
Symbol Span Test
Paper Folding Test
Scene Description Test
Boundary Extension Test

#### Test of premorbid functioning

This test^91^ provides estimates of IQ by asking participants to pronounce 70 irregularly spelt words. Two outcome measures are available.

*Raw score*. The number of correctly pronounced words.

*Estimate of full scale IQ*. The raw score on converted to an estimate of Full Scale IQ using the Test of Premorbid Functioning scorer.

#### Matrix reasoning test

This is a subtest of the Wechsler Adult Intelligence Scale IV [WAIS-IV^92^] designed to measure non-verbal problem solving and perceptual reasoning skills. Participants view an incomplete pattern or a series of abstract pictures and are required to select the correct option to fit the missing picture from a choice of possible options. Two outcome measure are available.

*Number correct*. The number of correct responses out of 26.

*Scaled score*. The number of correct responses is scaled given the participant’s age, providing a maximum score of 19. This is the standard outcome measure of the test.

#### Brixton spatial anticipation test

This test^93^ is a visuospatial sequencing task, testing a participant’s ability to detect rule changes. On each trial a participant is presented with an array of 10 circles (two rows of five), with one of the circles coloured blue. The position of the blue circle changes on each presentation and a participant is required to indicate where they believe the next blue circle will be, based on the pattern inferred from the previous presentations. Correct responses are those that follow the current pattern. Two outcome measures are available.

*Raw score*. The number of correct responses out of 54.

*Scaled score*. The number of correct responses is converted into a scaled score between 1 and 10. This is the typical outcome measure of the Brixton Spatial Anticipation Test.

#### F-A-S Test

This test^86^ is a measure of verbal fluency and requires participants to name as many words as they can (excluding proper nouns and repeating the same word with different endings) beginning with the selected letter F, A or S in one minute. The final score is the total number of words provided for the three letters.

#### Digit span test

This task is a memory span task from the WAIS-IV^92^ and measures working memory and verbal ability. Both forms of the Digit Span Test were used – forwards and backwards. For the forwards task, a participant is asked to repeat a number sequence (2–9 numbers in length, 2 sequences of each length) provided by the experimenter. For the backwards task, a participant is asked to repeat the provided number sequences (2 – 8 numbers in length) in the reverse order. Correct responses are those that match the sequence exactly. Four outcome measures are available.

*Forwards raw*. The number of correct responses to the forwards Digit Span Test, out of 16.

*Forwards scaled*. The number of correct responses to the forwards Digit Span Test converted to a scaled score dependent on the age of a participant. The maximum scaled score is 18.

*Backwards raw*. The number of correct responses to the backwards Digit Span Test out of 16.

*Backwards scaled*. The number of correct responses to the backwards Digit Span Test converted to a scaled score dependent on the age of a participant. The maximum scaled score being 19.

#### Symbol span test

This test is from the WMS-IV^87^ and assesses visual working memory. The principle follows that of the forwards Digit Span Test – participants are shown a series of abstract shapes (1–7 in length) which they are told to remember in that order. After seeing the order to be remembered, an array of abstract shapes is presented. Participants then have to select the correct shapes in the same order as they were first shown. Two outcome measures are provided.

*Raw score*. The number of correct responses out of 50.

*Scaled score*. A scaled score is calculated from the number of correct responses and the age of a participant. The maximum scaled score is 19.

#### Paper folding test

This test is taken from the Kit of Factor-Referenced Cognitive Tests^94^. The test measures a participant’s ability to manipulate or transform images of spatial patterns into different arrangements. As such, it is a formalized task of visuospatial mental imagery ability. A participant is shown a series of squares which represent a piece of paper being folded. In the final (fully folded) image a circle is drawn to show where the folded paper has had a hole punched through it. A participant then has to indicate, from 5 possible answers, what the unfolded piece of paper will look like – i.e. where the holes are located. Twenty questions are presented to a participant. The final score is the total number of correct responses.

### Scene description test

This test^95^ requires a participant to describe out loud a picture of a simple scene of a bench in park. A previously published principal components analysis^60^ showed that the Scene Description Test associated more with perceptual tests rather than with tests such as autobiographical memory recall and scene construction, which is why we have grouped it here with other general cognitive tests. Participants give descriptions until they came to a natural end or cannot add any more details. If required, a general probing protocol is utilized to elicit more details (e.g. is there anything else you can tell me?). All descriptions are audio recorded and transcribed for scoring. The scene description is split into statements which are then classified by the experimenter as belonging to one of four categories. Five outcome measures are available.

*Spatial references*. This refers to statements about the relative position of entities within the environment, directions relative to a participant’s vantage point, or explicit measurements (e.g. “to the left there is a…”).

*Entities present*. This category is a simple count of how many distinct entities (objects, people, animals) were mentioned (“I can see some birds”).

*Sensory descriptions*. This category consists of any statements describing (in any modality) properties of an entity as well as general weather and atmosphere descriptions.

*Thoughts/emotions/actions*. This category concerns any introspective thoughts or emotional feelings (“I have a sense of being alone”) as well as the thoughts, intentions, and actions of other entities in the scene (“he seems to be in a hurry”)

*Total content*. The total content score is the sum of the scores from the four categories.

#### Boundary extension test

Boundary extension occurs when individuals who are viewing scenes automatically imagine what might be beyond the view, and consequently later misremember having seen a greater expanse of the scene^96^. To test this phenomenon in the current study, a rapid serial visual presentation task was used. On each trial, a participant is presented with two pictures in rapid succession separated by a briefly visible visual noise mask (initial scene presentation = 250 ms; masked interstimulus interval = 250 ms). A participant then rates the second picture relative to the first, responding with one of five options “much closer up,” “a little closer up,” “the same”, “a little farther away,” or “much farther away,”. In some previous versions of this task, participants completed 24 trials, in which, unbeknownst to participants, all the pictures were exactly the same^95^. Here, eight additional picture pairs were included, four showing a second picture further away and four showing a second picture closer up (see Usage Notes for further discussion on this point). These trials were included only as controls and were not analysed. After each trial, a participant reported how confident they were about their decision on a three-point scale: 1 (not sure); 2 (fairly sure); 3 (very sure). Eleven outcome measures are available.

*Percentage much closer*. The percentage of much closer up responses made relative to the total number of responses made.

*Percentage little closer*. The percentage of a little closer up responses made relative to the total number of responses made.

*Percentage same*. The percentage of the same responses made relative to the total number of responses made.

*Percentage little farther*. The percentage of a little farther away responses made relative to the total number of responses made.

*Percentage much farther*. The percentage of much farther away responses made relative to the total number of responses made.

*Average confidence rating much closer*. Mean confidence rating for the much closer up responses.

*Average confidence rating little closer*. Mean confidence rating for the little closer up responses.

*Average confidence rating same*. Mean confidence rating for the same responses.

*Average confidence rating little farther*. Mean confidence rating for the little farther away responses.

*Average confidence rating much farther*. Mean confidence rating for the much farther away responses.

*Mean score*. A mean boundary extension score was calculated for each participant by averaging the response ratings (much closer up = −2; a little closer up = −1; the same = 0; a little farther away =  + 1; much farther away =  + 2) across all the trials. A mean score of ‘0’ reflects no boundary extension, while a minus score reflects the occurrence of boundary extension.

### Test order

The order of tests was arranged so as to minimize test interference, for example, not having a verbal test followed by another verbal test, and to provide visits of approximately equal length (~3–3.5 hr, including breaks). The cognitive tests were presented for each participant in the following order:

*Visit 1*: Concrete Verbal Paired Associates Test (learning), Warrington Recognition Memory Test for Scenes, Dead or Alive Test, Symbol Span Test, Scene Description Test, Concrete Verbal Paired Associates Test (delayed recall), Logical Memory Test (learning), Rey–Osterrieth Complex Figure Test (copy), Test of Premorbid Functioning, Warrington Recognition Memory Test for Faces, Brixton Spatial Anticipation Test, Logical Memory Test (delayed recall), Rey–Osterrieth Complex Figure Test (delayed recall), and Warrington Recognition Memory Test for Words.

*Visit 2*: Navigation Test, Abstract Verbal Paired Associates Test (learning, with delayed recall 30 min later). Note that this visit also included MRI scanning (further details of this are provided later).

*Visit 3*: Scene Construction Test, Future Thinking Test, Rey Auditory Verbal Learning Test (learning), Paper Folding Test, Digit Span Test, Matrix Reasoning Test, Rey Auditory Verbal Learning Test (delayed recall), Autobiographical Interview, WMS Verbal Paired Associates Test (learning), Object-Place Association Test, Boundary Extension Test, WMS Verbal Paired Associates Test (delayed recall), and F-A-S Test.

### Strategies

There is currently no standard methodology for studying strategy use in real world and laboratory-based memory tasks. We therefore designed a novel protocol for collecting and analysing detailed strategy information for a number of the specifically memory tasks that were of particular interest in this study. Details of this protocol have been published previously^64^ but are recapitulated here for the Reader’s convenience.

#### Identification of strategies

To identify possible strategies used to perform the tasks, 30 participants were recruited who did not take part in the main study (15 female; mean age 27.07 years, *SD* = 7.32). Participant recruitment was based on an individual’s general use of visual imagery. The use of visual imagery is a well-known strategy^33,97,98^ and we wanted to represent all types of strategies, not just those that are based on visual imagery. General visual imagery use was determined via the Spontaneous Use of Imagery Questionnaire^78^, where scores can range from 12 (very low/no spontaneous use of visual imagery) to 60 (high spontaneous use of visual imagery). The average score of the participants in this identification of strategies study was 40.03 (SD = 9.97) with a range from 24 to 57.

To collect information on individual task strategies, each participant first performed the real world and laboratory-based memory tasks, after which they were asked open-ended questions about the strategies they employed for each task. Participants were encouraged to report all strategies that they used for a task in as much detail as possible, regardless of how much or little they used them. Strategy responses from the participants were then combined with any relevant additional strategies identified from the extant literature. This provided a large pool of potential strategies, ranging from 12 to 24 strategies for each task.

For our purposes^64^, each strategy question was allocated to a group. Three main strategy groups were observed: Scene Visual Imagery strategies, Other Visual Imagery strategies and Verbal strategies. A Scene Visual Imagery strategy is one which evoked a visual image of a scene, i.e. the visual imagery had a sense of depth and background. An Other Visual Imagery strategy is one which evoked visual imagery, but this could not be defined as a scene. There was no sense of depth or background, a typical example being an image of a single object. A Verbal strategy is one which evoked no visual imagery at all, with reliance instead upon words and phrases.

Importantly, while each pool of strategies was specific to the task in question, they all contained the same three main strategy categories. This allowed for the collection of highly detailed strategy data that was comparable across multiple tasks.

#### Strategy questionnaires

The information from the strategy identification study was used to construct a strategy questionnaire for each task of interest. The questionnaires were participant-paced and led, but with the involvement of the experimenter where required. Strategy data were collected during the fourth and final visit to the Centre. Three steps were involved. First, a brief reminder of the task was presented. Second, participants selected the strategies they used for that task from an extensive list of possible strategies. Third, participants ranked their selected strategies in relation to their degree of use.

Strategies were obtained for all of the real-world tasks: Scene Construction Test, Autobiographical Interview (strategies were collected for each memory age of the Autobiographical Interview), Future Thinking Test and Navigation Test (both for the learning of the town and each of the five navigation tasks). In addition, strategy data were collected for all of the laboratory-based memory tasks, with the exception of the copy task of the Rey–Osterrieth Complex Figure Test. As such, strategy data was obtained for the delayed recall of the Rey–Osterrieth Complex Figure Test, the learning, immediate and delayed recall of the Object-Place Association Test, the learning, immediate and delayed recall of the Rey Auditory Verbal Learning Test, the learning, immediate and delayed recall of the Logical Memory Test, the learning, immediate and delayed recall of the WMS Verbal Paired Associates, the learning, immediate and delayed recall of the Concrete Verbal Paired Associates Test, the learning, immediate and delayed recall of the Abstract Verbal Paired Associates Test (note that the same strategies were used across the three verbal paired associate tasks to enable direct comparisons between them), the encoding and recognition tests of the Warrington Recognition Memory Tests for Words, Faces and Scenes, and the Dead or Alive Test.

*Task reminder*. The task reminder varied according to the task. For some tasks, a picture of the task was presented, while for others, the tasks were verbally described. The experimenter then ensured that the participant fully remembered the task (providing additional information if required) before the participant moved on to the strategy selection.

*Strategy selection*. Following the task reminder, all the possible strategies for the task that were reported in the strategy identification study were presented as a list on a computer screen. For each strategy, participants were requested to respond either “Yes” (that they used the strategy) or “No” (that they did not use the strategy). A response was required for every strategy to ensure that none were accidently overlooked. It was made clear that selecting one strategy did not preclude the selection of any of the others, as more than one strategy could be deployed during a task. For all tasks, the option “Other”, with space to describe a strategy not represented on the list, was also available.

For two of the strategy questions a Yes response resulted in participants being asked for further information. These questions were the Vague Visual strategy and Aural with Visual Imagery strategy. The Vague Visual strategy statement was: “*I had a very vague or fleeting sense of a visual image in my mind, which was really unclear and hazy – it was more the idea of an image in my mind than actually seeing an image itself clearly*”. If participants responded Yes to this statement, then they were asked to select which of four additional options best described the vague image used: *If you have selected YES for the above, please now select one of the options below that best describes your experience. My very vague impression is that the image: was scene-like (in that I had a sense of a space or context, albeit very vague); had multiple elements but was not a scene; involved single isolated objects; I cannot describe the image*. The Aural with Visual Imagery strategy statement was: “*I recalled a list of facts from the story, as though replaying an audio recording of the experiment, this then caused me to experience related visual imagery”*. If participants responded Yes to this statement, then they were asked to select which of two options best described the image experienced: “*The visual imagery that was evoked could be described as something scene-like (in that I had a sense of a space or context, albeit very vague)”* or *“The visual imagery that was evoked could be described as something comprising single objects (not a scene)”*.

*Strategy ranking*. After selecting the strategies, a list of the strategies that a participant indicated they used during the task was then presented to them. They were asked to rank each of the strategies according to how much of the time they used them. Outside of these instructions they were free to indicate any form of ranking. Thus, if they felt they used multiple strategies equally this could be indicated. For example, if three strategies were chosen they could be ranked:

1, 2, 3 – where the strategy ranked 1 was used most of the time, followed by the strategy ranked 2, and then the strategy ranked 3.

1, 1, 1 – where all strategies were used equally.

1, 2, 2 – where one strategy was used the most, and the other two less frequently, but the secondary strategies were used equally.

*Question order*. Tests were not probed in the order they were administered because in the strategies session, where many tests were being examined in one go, other considerations came into play. For example, a long test was often followed by a shorter test to aid concentration, and a visual test was followed by a verbal test and not by another visual test in order to minimize test interference. Half the participants followed this test order (order 1 – see below) and visual strategies were presented first for consideration for each task. For the other half of the participants, and to mitigate bias, the test order was reversed and verbal strategies were presented for consideration first for each task.

Order 1: Warrington Recognition Memory Test for Faces (encoding, then recognition), WMS Verbal Paired Associates Test (learning, immediate recall, delayed recall), Rey–Osterrieth Complex Figure Test (delayed recall), Concrete Verbal Paired Associates Test (learning, immediate recall, delayed recall), Autobiographical Interview (early childhood, teenage, adulthood, last year), Warrington Recognition Memory Test for Words (encoding, recognition), Object-Place Association Test (learning, immediate recall, delayed recall), Abstract Verbal Paired Associates Test (learning, immediate recall, delayed recall), Navigation Test (learning, clip recognition, scene recognition, proximity judgements, route knowledge, sketch map), Rey Auditory Verbal Learning Test (learning, immediate recall, delayed recall), Dead or Alive Test, Scene Construction Test, Future Thinking Test, WMS Verbal Paired Associates Test (learning, immediate recall, delayed recall), Warrington Recognition Memory Test for Scenes (encoding, recognition). The strategies were listed with visual imagery strategies first, followed by verbal strategies.

Order 2: as noted above, the task order was reversed, and the strategies were listed starting with verbal strategies first followed by visual imagery strategies.

### MRI data

Multimodal 3 T MRI data were collected from all participants over two scanning sessions (see Table 5 for a list of the neuroimaging data collected). Three MRI scanners were used to collect the data. All scanners were Siemens Magnetom TIM Trio systems with 32 channel head coils and were located at the same imaging centre, running the same software. The sequences were loaded identically onto the individual scanners. Participant set-up and positioning followed the same protocol for each scanner. A participant was only scanned on one scanner throughout.

**Table 5:** The MRI data.

MRI Data
Multi-Parameter Mapping
Diffusion Weighted Imaging
T2-Weighted High Resolution Structural Images
Manually Segmented Hippocampal Subfields
FLASH Structurals
Whole Brain Resting State
Partial Volume High Resolution Resting State

In the first scanning session, a participant underwent a FLASH Structural, Whole Brain Resting State, Partial Volume High Resolution Resting State and Multi-Parameter Mapping (MPM). The scanning session took 60 minutes. On a separate day, a participant underwent Diffusion Weighted Imaging (DWI), a second FLASH Structural and T2-Weighted High Resolution Structural Imaging. The scanning session took 67 minutes. Each sequence is described in detail below.

As well as all of the MRI data, we also make available the manual bilateral segmentations of the subfields along the full long axis of the hippocampus of 201 (of the 217) participants. Hippocampal subfield delineation was performed on the T2-Weighted High Resolution Structural Images by two researchers, and took approximately 8 hours per participant (see Segmentation of hippocampal subfields for further details).

#### MPM

MPM is a quantitative neuroimaging technique to model different properties of tissue microstructure^99–101^. Processing of these images using the hMRI toolbox^102^ results in four maps that are differentially (but not solely) sensitive to specific aspects of tissue microstructure. These are magnetisation transfer saturation (MT saturation), sensitive to myelination; proton density (PD), sensitive to tissue water content; the longitudinal relaxation rate (R_1_), sensitive to myelination, iron and water content (but primarily myelination); and the effective transverse relaxation rate (R_2_^*^), sensitive to tissue iron content.

For this dataset, whole brain MPM images were obtained at an isotropic resolution of 800 μm × 800 μm × 800 μm^99,101^. This protocol consisted of the acquisition of three multi-echo gradient acquisitions with either PD, T1 or MT weighting. Each acquisition had a repetition time (TR) of 25 ms. PD weighting was achieved with an excitation flip angle of 6°, which was increased to 21° to achieve T1-weighting. MT weighting was achieved through the application of a Gaussian RF pulse 2 kHz off resonance with 4 ms duration and a nominal flip angle of 220°. This acquisition had an excitation flip angle of 6°. The field of view (FOV) was 256 mm × 224 mm × 179 mm (Head-Foot × Anterior-Posterior (AP) × Right-Left (RL)). The multiple gradient echoes per contrast were acquired with alternating readout gradient polarity at eight equidistant echo times (TE) ranging from 2.34 to 18.44 ms in steps of 2.30 ms using a readout bandwidth of 488 Hz/pixel. Only six echoes were acquired for the MT weighted volume to facilitate the off-resonance pre-saturation pulse within the TR. To accelerate the data acquisition, partially parallel imaging using the GRAPPA algorithm was employed in each phase-encoded direction (AP and RL) with forty reference lines and a speed up factor of two. Calibration data (B0 and B1 images) were also acquired at the outset of each session to correct for inhomogeneities in the RF transmit field^103,104^. Scan time was 25 minutes.

#### DWI

DWI data were collected using the multiband accelerated echo planar imaging (EPI) pulse sequence developed by the Centre for Magnetic Resonance Research at the University of Minnesota [R012a-c, R013a on VB17, https://www.cmrr.umn.edu/multiband/^105,106^]. Acquisition parameters were: resolution = 1.7 mm isotropic; FOV = 220 mm × 220 mm × 138 mm; 60 directions with 6 interleaved B0 images, TE = 112 ms, TR = 4.84 s, with a multiband acceleration factor of 3. The sequence was performed 4 times – twice with B-values of 1000 and twice with B-values of 2500. The first acquisition of each set of B-values was performed with phase-encoding in the AP direction, the second in the PA direction. The total acquisition time was 22 min.

#### T2-Weighted high resolution structural images

T2-Weighted High Resolution Structural Images were obtained to permit hippocampal subfield delineation. Data were collected using a single-slab 3D T2-weighted turbo spin echo sequence with variable flip angles^107^ in combination with parallel imaging to simultaneously achieve a high image resolution of ~500 μm, high sampling efficiency, and short scan time while maintaining a sufficient signal-to-noise ratio. After excitation of a single axial slab, the image was read out with the following parameters: resolution = 0.52 × 0.52 × 0.5 mm, matrix = 384 × 328, partitions = 104, partition thickness = 0.5 mm, partition oversampling = 15.4%, FOV = 200 mm × 171 mm, TE = 353 ms, TR = 3,200 ms, GRAPPA × 2 in PE direction, bandwidth = 434 Hz/pixel, echo spacing = 4.98 ms, turbo factor in PE direction = 177, echo train duration = 881, averages = 1.9. For reduction of signal bias due to, for example, spatial variation in coil sensitivity profiles, the images were normalized using a pre-scan, and a weak intensity filter was applied as implemented by the scanner’s manufacturer. To improve the signal-to-noise ratio of the anatomical image used for segmentation, for 214 participants we acquired three scans each, with a total scanning time of 39 minutes. For three participants, two scans were obtained.

#### Segmentation of hippocampal subfields

We make available here the manual bilateral subfield segmentations of the full long axis of the hippocampus of 201 (of the 217) participants (i.e. 402 hippocampi). Due to low image quality, segmentation was not possible for the other 16 participants.

For each participant, the T2-weighted scans underwent Rician noise estimation^108^ and were then denoised using oracle-based discrete cosine transform [ODCT^109^], with additional denoising then applied to the ODCT denoised image using a prefiltered rotationally invariant nonlocal means filter^109^. This computed a single denoised image for each high resolution structural image. The denoised images were then co-registered and averaged to provide a final image for hippocampal segmentation for each participant.

Hippocampal segmentation was performed according to the methodology described by Dalton *et al*.^58^ using the ITK Snap software version 3.2.0. Masks were created for the following six subregions: DG/CA4, CA2/3, CA1, subiculum, pre/parasubiculum and uncus (see Fig. 1). Subfield segmentations were performed by two researchers. To assess inter-rater reliability, each researcher independently segmented both hippocampi of the same 20 participants (10% of the total) and analyses for each subfield were conducted using the Dice overlap metric^110^ and interclass correlation coefficients – see Technical validation for details.

#### FLASH structurals

Two whole brain 3D FLASH structural scans were acquired for each participant, one in each scanning session. Each acquisition had a resolution of 1 × 1 × 1 mm, TR = 10 ms, TE = 3.54 ms, flip angle = 10°, FOV = 240 mm × 256 mm × 176 mm. The acquisition time for each scan was three minutes.

#### Whole brain resting state

EPI images were obtained using scanning parameters optimized for reducing susceptibility-induced signal loss in the medial-temporal lobe: 48 transverse slices angled at −30°, TR = 3.36 s, TE = 30 ms, resolution = 3 × 3 × 3 mm, matrix size = 64 × 74, z-shim gradient moment of −0.4 mT/m ms^111^. Two hundred and twenty volumes (including the dummy volumes necessary to reach steady state) were acquired with the scan lasting approximately 13 min.

To enable correction of the distortions in the EPI images, B0-field maps (consisting of magnitude and phase images) were acquired with a standard manufacturer’s double-echo gradient-echo field map sequence. Acquisition parameters were: short TE = 10 ms, long TE = 12.46 ms, 64 axial slices with 2 mm thickness and 1 mm gap yielding whole brain coverage; in-plane resolution 3 × 3 × 3 mm, TR = 1,020 ms.

#### Partial volume high resolution resting state

Data were acquired using a 3D EPI sequence which has been demonstrated to yield improved BOLD sensitivity compared to 2D EPI acquisitions^112^. Image resolution was 1.5 × 1.5 × 1.5 mm and the FOV was 192 mm^2^ in-plane. Forty slices were acquired with 20% oversampling to avoid wrap-around artefacts due to the imperfect slab excitation profile. The TE was 37.30 ms and the TR was 3.65 s. Parallel imaging with GRAPPA image reconstruction^113^ acceleration factor 2 along the phase-encoding direction was used to minimize image distortions and yield optimal BOLD sensitivity. Two hundred volumes were acquired, including the dummy volumes necessary to reach steady state and the GRAPPA reconstruction kernel^112^, with the scan lasting approximately 13 min.

To enable correction of the distortions in the EPI images, B0-field maps (consisting of magnitude and phase images) were acquired with a standard manufacturer’s double-echo gradient-echo field map sequence. Acquisition parameters were: short TE = 10 ms, long TE = 12.46 ms, 64 axial slices with 2 mm thickness and 1 mm gap yielding whole brain coverage; in-plane resolution 3 × 3 × 3 mm, TR = 1,020 ms.

## Data Records

All data can be found in the Dryad data repository: 10.5061/dryad.2v6wwpzt3^114^. The cognitive data are available as Microsoft excel files. Raw MRI data have undergone DICOM conversion in the program Statistical Parametric Mapping (SPM; www.fil.ion.ucl.ac.uk/spm) and are provided as NifTI files (extension.*nii*). MRI data are available in zipped folders grouped by scan type, with each participant’s scans for that scan type in individual folders. This data organisation mirrors that of the Cambridge Centre for Ageing and Neuroscience (Cam-CAN) data repository^16^, the rationale being that data grouped by scan type means that users can avoid having to download all of the MRI data if they are only interested in one specific type of scan.

Multiple steps have been taken to preserve participant anonymity. Personally identifiable information (such as a participant’s name, date of birth, and contact information) has been deleted from all data. Using *spm_deface*, facial characteristics have been removed from anatomical MRI images (MPM images, T2-Weighted High Resolution Structural Scans, FLASH Structural scans and Fieldmap magnitude data). The resulting defaced images were visually inspected to confirm that defacing was successful and to ensure that no brain tissue was lost. Information about the time and date of a scan has also been removed.

### Sample demographics

The demographics of the sample can be found in the “Full_Results” excel file. Data are located on the first sheet called “Demographics”. Column headers (in row 1) provide details of the measure name and the coding used for scoring.

### Questionnaires

Questionnaire data are available in both summary form and item-by-item. Summary data are available in the “Full_Results” excel file, located on the second sheet called “Questionnaires”. These data are the standard outcome measure of each of questionnaire and their subscales (where applicable). The questionnaires are listed in alphabetical order.

Item-by-item data, that is, the response to each question for each questionnaire, are provided in the “Questionnaires_Item_by_Item” excel file. Each questionnaire has an individual sheet, and they are listed in alphabetical order. The questions for each questionnaire are provided in the order the questions were presented to participants. Where relevant, the subscale to which the question belongs (shown in bold), and whether the item requires reverse scoring, are also detailed. Coding information for categorical items (e.g. True or False) is indicated in the column headings.

### Real-world tests

Real-world data are available in summary form for all tests, and for each trial individually for the Scene Construction Test, the Autobiographical Interview and the Future Thinking Test.

Summary data are available in the “Full_Results” excel file, located on the third sheet called “Real-World Tests”. For the Scene Construction Test, the Autobiographical Interview and the Future Thinking Test, these data are the average performance for each measure across the whole task (i.e. across the seven imagined scenes, the five recalled autobiographical memories and the three imagined future scenarios). For Navigation, performance on each of the subtests described in the previous section is provided.

Trial level data are also available for the Scene Construction Test, the Autobiographical Interview and the Future Thinking Test in the “Real_World_Tests_Individual_Trials” excel file. Each test has an individual sheet. Within each sheet, the performance scores for each trial are provided in the order they were presented to participants. For the Scene Construction Test this was: the swimming pool of a luxury hotel, a busy fishing harbour, an old library, the boardroom of a big corporation, the ruins of a derelict building, a large circus tent, the inside an ancient cathedral. For the Autobiographical Interview it was: early childhood (up to age 11), teenage years (aged from 11–17), adulthood (age from 18 to 12 months prior to the interview; two memories were requested, these are shown in the order provided by the participant) and the last year (a memory from the last 12 months). For the Future Thinking Test the order was: an event at the weekend, next Christmas, the next time they will meet a friend.

### Laboratory-based memory tests

Laboratory-based memory task data are available in the “Full_Results” excel file, located on the fourth sheet called “Laboratory Memory Tests”. Data are provided for each of the outcome measures detailed in the Methods.

### Laboratory-based general cognitive tasks

Laboratory-based general cognitive task data are available in the “Full_Results” excel file, located on the fifth sheet called “Laboratory Cognitive Tests”. Data are provided for each of the outcome measures detailed in the Methods.

### Strategies

The strategy data are provided in the “Strategies” excel file. Each test for which strategies were obtained is shown on a separate sheet, listed in the same order as presented in the Methods. Participants could perform the strategies in one of two orders (see Methods), the order followed by the participant is detailed on each sheet. The strategy questions are listed in the order seen by participants, shown here using order 1.

For each test, the response to each strategy statement (i.e. did a participant use that specific strategy) is detailed first. A Yes response is coded as 1, a No response is coded as 0. The strategy category assigned for our research purposes (Scene Visual Imagery, Other Visual Imagery, Verbal) is detailed for each strategy statement. For the Vague Visual and Aural with Visual Imagery strategy statements, the responses to the more specific follow up options, rather than the initial more general statement (see Methods) are provided. Note that a No response to the initial statement means that a No response has been recorded for all the follow up options, while if a Yes response was made to the initial statement, the specific follow up option to which the Yes applied is detailed.

Following this, the rankings for each strategy are provided. Only strategies that the participant indicated using were ranked. Full details regarding the rankings are outlined in the Methods. In short, strategies not used are labelled as 0, then from 1 upwards a low number indicates high usage while high numbers indicate low usage. Rankings for the Vague Visual and Aural with Visual Imagery strategies (regardless of which follow up statement was selected) are under the general headings (Vague Visual or Aural with Visual Imagery).

### MRI data

#### MPM

MPM data are available for each participant in the zipped folders “MPM_1”, containing data from participants 1 to 108 and “MPM_2”, containing data from participants 109 to 217. Each participant has six subfolders within their main MPM folder. The “MPM_B1_Map” folder contains 22 images for B1 mapping. The “MPM_Fieldmap_Magnitude” folder and “MPM_Fieldmap_Phase” folder contain 2 and 1 images respectively for B0 calibration. The “MPM_T1_weighted” folder contains the 8 T1-weighted images, the “MPM_PD_weighted” folder has the 8 PD weighted images, and the “MPM_MT_weighted” folder contains the 6 MT weighted images.

#### DWI

DWI data are available for each participant in the zipped folder “DWI”. Each participant has four subfolders within their main DWI folder, each contacting 66 files. The folder “DWI_AP_B1000” contains the images with B-values of 1000 collected in the anterior to posterior direction. The folder “DWI_PA_B1000” contains the images with B-values of 1000 collected in the posterior to anterior direction. The “DWI_AP_B2500” folder contains the images with B-values of 2500 collected in the anterior to posterior direction. The final “DWI_PA_B2500” folder contains the images with B-values of 2500 collected in the posterior to anterior direction.

#### T2-Weighted high resolution structural images and hippocampal subfield segmentation

The high resolution structural images and hippocampal subfield segmentations are available in the zipped folder “Hippocampal Subfield Segmentation”, which contains three subfolders – “Main Sample”, “DICE” and “Low Quality Scans”.

The “Main Sample” folder contains the data of the 201 participants for whom hippocampal subfield segmentations could be performed. For each participant, we provide the defaced high resolution structural images (typically 3), the denoised and averaged structural image used for hippocampal segmentation (e.g. anon_meansMXXXXXX-0008-00001-000001-01_denoised_prinlm_b2.0.nii) and the bilateral hippocampal subfield segmentation itself (Hippocampal_Segmentation.nii).

The “DICE” folder contains the data of the 20 participants used for inter-rater reliability. For each participant the hippocampal subfield segmentation performed for inter-rater reliability (DICE_Hippocampal_Segmentation.nii) is provided. Note that all subfield segmentations in the “DICE” folder were performed by segmenter IAC, with the subfield segmentations for the same participants in the “Main Sample” folder being performed by segmenter MAD. In addition, and for the User’s convenience, each participant’s folder in the “DICE” folder also contains the defaced high resolution structural images and the averaged and denoised structural image used for hippocampal segmentation – these images being the same as those provided in the “Main Sample” folder for that participant.

The “Low Quality Scans” folder contains the data of the 16 participants whose high resolution structural scans (even after denoising and averaging) were deemed too poor for hippocampal subfield segmentation to be performed. For each participant we provide the defaced high resolution structural images (typically 3) and the defaced averaged and denoised structural image considered not suitable for subfield segmentation.

Summary data for the hippocampal subfield segmentations are provided in the excel file “Hippocampal_Subfield_Volumes”. This contains details of the segmenter who performed the segmentation included in the Main Sample (either IAC or MAD), a quality rating for each participant’s denoised and averaged structural image used for segmentation, and the volumes for each subfield as provided by ITK snap (v 3.2). The scan quality rating is provided on a three-point scale: 1 (Good); 2 (Acceptable); 3 (Difficult). The majority of the images (167) were rated Good, with 23 rated Acceptable and 11 regarded as Difficult. Subfield volumes are provided as bilateral, left, right, anterior (bilateral) and posterior (bilateral). In line with the literature^115,116^, the anterior was defined as proceeding from the first slice where the hippocampus can be observed in its most anterior extent until the final slice of the uncus (the uncal apex), and the posterior hippocampus was defined from the first slice following the uncal apex until the final slice of observation in its most posterior extent.

The text file “Hippocampal Subfield Segmentation Labels.txt”, is the ITK snap label description file – providing the label information for the hippocampal segmentations when opened in ITK snap.

#### FLASH structurals

The FLASH structural data collected in the first MRI session are available in the folder “FLASH Structural Session 1” and the FLASH structural data collected in the second MRI session are available in the folder “FLASH Structural Session 2”. Two files are available for each participant for each session. The file with the lower number (e.g. anon_sMXXXXXX-000**2**-00001-000176-01.nii) is the anatomical image, and the file with the higher number (e.g. anon_sMXXXXXX-000**3**-00001-000176-01.nii) is the phase image.

We suggest using the FLASH structural data that correspond to the same session as the main modality of interest (i.e. using FLASH Structural Session 1 with the Whole Brain Resting State and Partial Volume High Resolution Resting State data, and FLASH Structural Session 2 with the T2-Weighted High Resolution Structural Images).

#### Whole brain resting state

The Whole Brain Resting State images and associated B0 field maps are in the folder “Whole Brain Resting State”. Each participant has three subfolders. The “Whole_Brain_Resting_State” folder contains the 220 EPI images. The “WB_Fieldmap_Magnitude” folder and “WB_Fieldmap_Phase” folder contain 2 and 1 images respectively for the B0-field map to enable correction of the distortions in the EPI images.

#### Partial volume high resolution resting state

The Partial Volume High Resolution Resting State images and associated B0 field maps can be found in the folder “Partial Vol Hi Res Resting State”. Within this folder, each participant has three subfolders. The “Partial_Volume_Resting_State” folder contains the 200 partial volume EPI images. The “PV_Fieldmap_Magnitude” folder and “PV_Fieldmap_Phase” folder contain 2 and 1 images respectively for the B0-field map to enable correction of the distortions in the EPI images.

## Technical Validation

### Questionnaires

Questionnaire data were collected online, and included a built-in requirement to respond to every question, thus eschewing missing data.

Across the suite of questionnaires deployed there was overlap in terms of their relationship with a particular cognitive process. This allowed for validation across questionnaires, because overlapping questionnaires should be correlated with each other, which was indeed the case. For example, within the imagery-based questionnaires, the OSIVQ Object-Scene subscale correlated with the PSIQ Appearance subscale (r = 0.55, p < 0.001) and the SUIS (r = 0.60, p < 0.001), and the PSIQ Appearance subscale and the SUIS were also correlated (r = 0.43, p < 0.001). Within the memory questionnaires, the overall score of the MEQ correlated both with the SMQ (r = 0.46, p < 0.001) and SAM Episodic subscale (r = 0.61, p < 0.001), and the SMQ and SAM Episodic subscale were also correlated (r = 0.63, p < 0.001). Similar correlations were observed within questionnaires assessing navigation, with the Santa Barbara Sense of Direction Scale and SAM Spatial subscale being highly correlated (r = -0.80, p < 0.001). We note that high subjective ratings of navigation ability are reflected in low scores on the Santa Barbara Sense of Direction Scale and high scores on the SAM Spatial subscale, hence the negative correlation. Finally, the Verbaliser and OSIVQ Verbal subscale, two questionnaires assessing verbal processing, were also well correlated (r = 0.69, p < 0.001).

Also of note is one of the research questions we addressed with this data set concerning the relationship between some of the questionnaires and the real world test data^61^. We identified, as predicted, significant relationships between imagery questionnaires and the Scene Construction Test, and navigation questionnaires and the Navigation Test. The same published paper also provides initial validation of the One Sentence Questionnaire, which we found to produce similar results as the longer well-established questionnaires.

Taken together, these analyses suggest that the questionnaire data are high quality.

### Real-world tests

All experimenters underwent extensive training before administering the real-world tests. Consistent administration both across experimenters and time (given the two year data collection period) was ensured by all experimenters following the same detailed protocols. Random spot-checks were also carried out throughout data collection by senior team members.

Validation of real world test scoring was undertaken by double scoring 20% of the data using the most stringent approach to identifying across-experimenter agreement, namely an inter-class correlation coefficient, with a two way random effects model looking for absolute agreement. Details of the double scoring for each task are provided in the sections below. For reference, an inter-class correlation coefficient between 0.75 and 0.90 is considered good reliability, and an inter-class correlation coefficient of 0.90 or above is considered excellent reliability^117^. In addition, as scoring was performed manually, all data were counted and entered by two experimenters to reduce data entry errors.

The real-world data have been utilised in 6 studies^52,59–63^. One examined the relationship between the ability to imagine scenes, recall the past and imagine future experiences^60^ and another investigated the relationship between the real world test data and self-report questionnaires^61^. Studies were also performed to investigate the relationship between real world task performance and hippocampal grey matter volume^63^ and hippocampal grey matter tissue microstructure^59^, between autobiographical memory recall ability and the MR g-ratio^62^ and between autobiographical memory recall ability and hippocampal subfield grey matter volume^52^.

#### Scene construction test

Double scoring was performed on the data of 20% of the participants (i.e. 44 participants, which equates to 308 individual scenes). As scenes were scored by four different experimenters, double scoring was performed on 20% of each experimenter’s scoring. Inter-class correlation coefficients were calculated for each content score and for the quality ratings. This was performed both for individual scenes and as an average of all seven scenes across each participant. All inter-class correlation coefficients were above 0.9 (full details in Table 6).

**Table 6:** Double scoring of the Scene Construction Test.

	Rating				
	Spatial References	Entities Present	Sensory Descriptions	Thoughts/Emotions/Actions	Quality Ratings
**For each individual scene**					
n = 308	0.90	0.96	0.94	0.90	0.90
**For each individual participant (i.e. the score is averaged across the seven scenes)**					
n = 44	0.91	0.99	0.97	0.91	0.93

#### Autobiographical interview

Double scoring was performed on the data of 20% of the participants (i.e. 44 participants, which equates to 215 individual memories). Memories were scored by three experimenters, with double scoring performed on 20% of each experimenter’s scoring. Inter-class correlation coefficients were calculated for each internal detail category and the overall internal total metric, as well as each external detail category and the overall external total score. This double scoring was performed both for individual memories and as an average of all five memories across each participant. For internal details the majority of inter-class correlation coefficients were above 0.9, with the lowest at 0.81 (full details in Table 7). For external details the majority of inter-class correlation coefficients were around 0.8 (full details in Table 8). External Events had the lowest inter-class correlation coefficients at 0.73 and 0.74, likely due to their very low occurrence (mean, across the participants, for the five recalled memories = 0.2, SD = 0.35). For the experimenter ratings, the majority of inter-class correlation coefficients were above 0.9, with the lowest at 0.79 (full details in Table 9).

**Table 7:** Double scoring of the Autobiographical Interview – Internal Details.

	Rating					
	Internal Event	Internal Place	Internal Time	Internal Perceptual	Internal Emotion	Internal Total
**For each individual memory**						
n = 215	0.92	0.85	0.94	0.92	0.86	0.94
**For each individual participant (i.e. the score is averaged across the five memories)**						
n = 44	0.95	0.88	0.96	0.94	0.81	0.97

**Table 8:** Double scoring of the Autobiographical Interview – External Details.

	Rating				
	External Event	External Semantic	External Repetition	External Other	External Total
**For each individual memory**					
n = 215	0.73	0.80	0.79	0.78	0.84
**For each individual participant (i.e. the score is averaged across the five memories)**					
n = 44	0.74	0.84	0.82	0.82	0.87

**Table 9:** Double scoring of the Autobiographical Interview – Experimenter Ratings.

	Rating					
	Episodic Richness	Time	Place	Perceptual	Emotion	Time Integration
**For each individual memory**						
n = 215	0.86	0.92	0.87	0.90	0.84	0.79
**For each individual participant (i.e. the score is averaged across the five memories)**						
n = 44	0.90	0.94	0.90	0.96	0.84	0.80

#### Future thinking test

Double scoring was performed on the data of 20% of the participants (i.e. 44 participants, which equates to 132 individual future scenes). Future scenes were scored by four experimenters, with double scoring performed on 20% of each experimenter’s scoring. Inter-class correlation coefficients were calculated for each content score and for the quality ratings. This was performed both for individual future scenes and as an average of all three future scenes across each participant. The majority of inter-class correlation coefficients were above 0.9, with the lowest at 0.88 (full details in Table 10).

**Table 10:** Double scoring of the Future Thinking Test.

	Rating				
	Spatial References	Entities Present	Sensory Descriptions	Thoughts/Emotions/Actions	Quality Ratings
**For each individual future scene**					
n = 132	0.90	0.94	0.93	0.88	0.90
**For each individual participant (i.e. the score is averaged across the three future scenes)**					
n = 44	0.94	0.95	0.96	0.88	0.92

#### Navigation sketch map

Navigation Sketch Maps were scored by three experimenters, with double scoring performed on 20% of each experimenter’s scoring (n = 42 maps). Inter-class correlation coefficients were calculated for each map category and the two experimenter ratings. All the inter-class correlation coefficients were above 0.89 (full details in Table 11).

**Table 11:** Double scoring of the Navigation Sketch Maps.

	Rating					
	Road Segments	Road Junctions	Number of Landmarks	Landmark Placement	Map Orientation	Map Categorisation
n = 42	0.95	0.96	0.97	0.96	0.96	0.89

### Laboratory-based memory and general cognitive tests

As with the real world tests, extensive training was provided to all experimenters before administering the laboratory-based memory and laboratory-based general cognitive tests. This was supplemented by the provision of detailed protocols which were followed by each experimenter. Random spot-checks were also carried out throughout data collection by senior team members.

Where possible, tests were computerised to reduce the potential for variations in data collection and errors in data scoring and entry. For tests performed and scored manually, data were counted and entered by two experimenters to reduce data entry errors. Conversion of raw scores to scaled scores was performed by automated scripts. These scripts were checked by multiple experimenters to ensure exact agreement with the published protocols.

For the Scene Description Test, double scoring was performed on the data of 20% of the participants (i.e. 43 participants). Scenes were scored by three experimenters, with double scoring performed on 20% of each experimenter’s scoring. Inter-class correlation coefficients were calculated for each content score. All inter-class correlation coefficients were above 0.85 (full details in Table 12).

**Table 12:** Double scoring of the Scene Description Test.

	Rating			
	Spatial References	Entities Present	Sensory Descriptions	Thoughts/Emotions/Actions
n = 43	0.88	0.91	0.93	0.85

One of the research questions addressed with this data set investigated the relationships between the laboratory-based tasks^60^. Within this published paper we demonstrated that, as would be expected, performance on tests thought to assess particular cognitive processes were grouped together in a principal component analysis. For example, tasks assessing executive function (Matrix Reasoning Test, F-A-S Test, Digit Span Test, Symbol Span Test) comprised one component, while those assessing verbal recall (Rey Auditory Verbal Learning Test, Logical Memory Test, WMS Verbal Paired Associates Test, Concrete Verbal Paired Associates Test, Abstract Verbal Paired Associates Test) were grouped in another component. Overall, these findings suggest the data are reliable.

### Strategies

Strategy data were collected using computerised online forms when participants visited our Centre, and included a built-in requirement to respond to every question, thus eschewing missing data. In addition, the presence of an experimenter also ensured that participants understood the instructions, completed the questionnaires accordingly, remembered all of the tasks for which strategies were being obtained, and allowed the participant to ask questions of the experimenter at any time. Experimenters also encouraged participants to take breaks to ensure attention remained high throughout.

An examination of the strategy data from the Scene Construction Test, Autobiographical Interview, Future Thinking Test, the Navigation Tests, Concrete Verbal Paired Associates Test, Abstract Verbal Paired Associates Test and the Dead or Alive Test has been published^64^. High quality data were identified for all of these tasks. Furthermore, ‘Other’ descriptions were rarely provided by participants, and there was no situation where the Other description referred to new strategies that were not already represented on the list of strategies already available. This suggests that the strategy protocol did not omit any key strategies.

### MRI data

#### MPM

The MPM data have been utilised in four published studies^59,62,63,65^, one focusing on the B1 and B0 maps^65^, another investigating grey matter volume using the MT saturation map^63^, one assessing grey matter tissue microstructure properties using MT saturation, PD R_1_ and R_2_^*^ maps^59^, and one utilising the white matter of the MT saturation map (in combination with the DWI data) to calculate the MR g-ratio^62^.

Across these studies, the MPM data were successfully processed using the hMRI toolbox^102^, segmented into grey and white matter probability maps, analysed in widely-used MNI space, and co-registered with other imaging modalities.

In addition, we also examined basic properties of the tissue microstructure maps to test whether they were in line with expectations. In healthy individuals, myelination levels have been reported to be higher in primary sensory regions^118,119^, while iron levels are increased in the substantia nigra and red nucleus^120,121^. We found that, as expected, higher MT saturation values (reflecting higher myelination) were identified in Heschl’s gyrus (mean MT = 0.90, SD = 0.035) compared to the mean of the grey matter (mean MT = 0.88, SD = 0.031; t(216) = 15.90, p < 0.001), and that higher R_2_^*^ values (reflecting higher iron) were observed in the substantia nigra (mean R_2_^*^ = 26.76, SD = 3.21; t(216) = 48.03, p < 0.001) and red nucleus (mean R_2_^*^ = 23.95, SD = 3.05; t(216) = 35.28, p < 0.001) compared to the mean of the grey matter (mean R_2_^*^ = 17.03, SD = 0.63). Taken together, these analyses suggest that the MPM data are high quality.

#### DWI

The DWI data have been used in two published studies^62,66^, one outlining a new pipeline to reduce susceptibility distortion related image blurring for diffusion MRI data analysed using SPM^66^, and the other investigating the relationship between the MR g-ratio, and other white matter microstructure tissue properties, and autobiographical memory recall ability^62^. Across these two studies, the diffusion data have been fully pre-processed, undergone diffusion tensor^122^, diffusion kurtosis^123^ and neurite orientation dispersion and density imaging [NODDI^124^] fitting, co-registered with MT saturation maps, and transformed and analysed in MNI space. No excessive movement or artefacts were identified when performing these analyses, suggesting the DWI data are high quality.

#### T2-Weighted high resolution structural images and hippocampal subfield segmentation

Our primary goal for collecting the T2-Weighted High Resolution Structural Images was to perform manual segmentation of hippocampal subfields. Segmentation requires high quality data to identify the landmarks used for delineation of the subfields, and this was available for 201 of the 217 participants. The data for the 16 participants deemed unsuitable for hippocampal segmentation are provided separately for completeness, in case they may be of sufficient quality for other uses. Hippocampal subfield segmentation was performed by two segmenters – IAC and MAD, MAD being the first author of the hippocampal subfield segmentation protocol that was followed^58^.

Reliability of the hippocampal subfield segmentations was assessed using inter- and intra-rater reliability measures. Our main focus was on inter-rater reliability, with each researcher independently segmenting both hippocampi of the same 20 participants (10% of the total). As hippocampal segmentation took approximately 8 hours per participant, subfield segmentation of the full sample was performed over a period of 3.5 years (from July 2018 to December 2021). Independent hippocampal segmentations to assess reliability were performed throughout this time period, serving also to provide a measure of consistency over time; were either researcher to deviate from the segmentation protocol over the 3.5 years, then we would know this because inter-rater measures would reduce.

Reliability analyses were performed for each subfield using the Dice overlap metric^110^ which produces a score between 0 (no overlap) and 1 (perfect overlap). Inter-class correlation coefficients were also computed (using a two way random effects model looking for absolute agreement), where inter-class correlation coefficients between 0.75 and 0.90 are considered to have good reliability and inter-class correlation coefficients of 0.90 or above are considered to have excellent reliability^117^.

Inter-rater reliability metrics were equivalent to those reported in the extant literature^48,51,56,125–129^. Dice inter-rater reliability was 0.85 for DG/CA4, 0.68 for CA2/3, 0.78 for CA1, 0.80 for subiculum, 0.70 for pre/parasubiculum and 0.83 for the uncus. Inter-class correlation coefficients were 0.91 for DG/CA4, 0.75 for CA2/3, 0.91 for CA1, 0.90 for subiculum, 0.76 for pre/parasubiculum and 0.97 for the uncus. The results for each of the 20 individual Dice inter-rater reliability analyses (including the date of each segmentation) are provided in Table 13. At all time points, Dice inter-rater reliability scores were in line with previous time points and publications, demonstrating high levels of consistency over the 3.5 year period, as well as high reliability between the two segmenters.

**Table 13:** Individual Dice inter-rater reliability results for the 20 reliability hippocampal subfield segmentations, presented in date order of segmentation.

Subfield	Participant: 146 IAC: Aug-18 MAD: July-18	Participant: 15 IAC: Sept-18 MAD: July-18	Participant: 24 IAC: Sept-18 MAD: July-18	Participant: 8 IAC: Sept-19 MAD: May-20	Participant: 13 IAC: Sept-19 MAD: May-20
DG/CA4	0.85	0.86	0.87	0.84	0.83
CA2/3	0.70	0.70	0.73	0.70	0.63
CA1	0.79	0.79	0.77	0.80	0.78
Subiculum	0.80	0.79	0.79	0.82	0.79
Pre/para	0.75	0.72	0.69	0.75	0.68
Uncus	0.85	0.82	0.81	0.82	0.80
	**Participant: 148** **IAC: March-20** **MAD: May-20**	**Participant: 9** **IAC: April-20** **MAD: May-20**	**Participant: 156** **IAC: April-20** **MAD: May-20**	**Participant: 173** **IAC: May-20** **MAD: June-20**	**Participant: 111** **IAC: June-20** **MAD: June-20**
DG/CA4	0.83	0.83	0.84	0.85	0.84
CA2/3	0.69	0.65	0.68	0.63	0.69
CA1	0.80	0.78	0.79	0.79	0.79
Subiculum	0.80	0.80	0.81	0.78	0.81
Pre/para	0.70	0.70	0.75	0.67	0.70
Uncus	0.81	0.84	0.86	0.82	0.85
	**Participant: 188** **IAC: July-20** **MAD: Sept-20**	**Participant: 196** **IAC: Nov-20** **MAD: Nov-20**	**Participant: 198** **IAC: Nov-20** **MAD: Nov-20**	**Participant: 216** **IAC: Nov-20** **MAD: Nov-20**	**Participant: 37** **IAC: Feb-21** **MAD: Feb-21**
DG/CA4	0.88	0.86	0.87	0.86	0.87
CA2/3	0.71	0.67	0.60	0.69	0.66
CA1	0.82	0.80	0.73	0.77	0.82
Subiculum	0.82	0.80	0.77	0.83	0.82
Pre/para	0.68	0.72	0.70	0.70	0.71
Uncus	0.82	0.84	0.84	0.82	0.85
	**Participant: 110** **IAC: March-21** **MAD: April-21**	**Participant: 128** **IAC: May-21** **MAD: June-21**	**Participant: 89** **IAC: July-21** **MAD: July-21**	**Participant: 10** **IAC: Oct-21** **MAD: July-18**	**Participant: 94** **IAC: Oct-21** **MAD: July-18**
DG/CA4	0.83	0.86	0.86	0.83	0.86
CA2/3	0.66	0.67	0.70	0.70	0.65
CA1	0.78	0.76	0.78	0.76	0.79
Subiculum	0.80	0.80	0.82	0.78	0.79
Pre/para	0.68	0.67	0.70	0.68	0.70
Uncus	0.84	0.84	0.85	0.82	0.82

*Note*. The two segmenters were IAC and MAD; Pre/para = Pre/parasubiculum.

Intra-rater reliability was also high for both segmenters. For IAC, Dice intra-rater reliability (over 5 participants, measured on average 32.8 months (SD = 13.22) apart) was 0.91 for DG/CA4, 0.81 for CA2/3, 0.86 for CA1, 0.86 for subiculum, 0.80 for pre/parasubiculum and 0.89 for the uncus, with inter-class correlation coefficients of 0.96 for DG/CA4, 0.93 for CA2/3, 0.85 for CA1, 0.99 for subiculum, 0.83 for pre/parasubiculum and 0.97 for the uncus. For MAD (as previously reported in^58^), Dice intra-rater reliability (over 6 participants, measured 3 months apart) was 0.86 for DG/CA4, 0.76 for CA3/2, 0.85 for CA1, 0.86 for subiculum, 0.75 for pre/parasubiculum and 0.87 for the uncus, with inter-class correlation coefficients of 0.91 for DG/CA4, 0.84 for CA2/3, 0.89 for CA1, 0.95 for subiculum, 0.72 for pre/parasubiculum and 0.96 for the uncus.

One study has used the full set of hippocampal subfield segmentations to investigate the relationship between hippocampal subfield volume and autobiographical memory recall ability^52^. A second study^47^ used 15 of the subfield segmentations (participant numbers: 10, 15, 24, 35, 48, 51, 60, 94, 117, 146, 155, 167, 179, 181, 208) to investigate differences in the functional connectivity of the hippocampal subfields between younger and older participants.

#### FLASH structurals

The two FLASH Structural scans were visually inspected for artefacts and defacing issues, and none were found.

#### Whole brain resting state

The Whole Brain Resting State data have not been used in a published study to date. However, the extensive technical validation performed for the other MRI data suggest a high level of data quality for these participants. In addition, the MRI sequence used to collect the whole brain resting state data has provided high quality data in other samples collected at our Centre, which have been examined using multiple analysis techniques^130–135^. We would expect, therefore, these data to be of a similar high quality.

#### Partial volume high resolution resting state

Fifteen of the Partial Volume High Resolution Resting State datasets have been used in a published study^47^. For these participants (participant numbers: 10, 15, 24, 35, 48, 51, 60, 94, 117, 146, 155, 167, 179, 181, 208), the EPI images were corrected for distortions using the B0-field maps and co-registered to the whole brain FLASH Structural scan. The data were analysed using the hippocampal subfield ROIs from hippocampal subfield segmentations performed on the T2-weighted images to investigate the functional connectivity along the anterior–posterior axis of hippocampal subfields. No issues were identified during any analysis step. While the Partial Volume High Resolution Resting State data of the other participants have not been investigated in detail, we expect them to be of a similar high quality.

## Usage Notes

### Scene construction test, autobiographical interview and future thinking test

Data protection legislation and the conditions of our Ethics Committee approval prevent us from sharing the original audio or transcribed descriptions of scenes, memories or future imaginings. As outlined above, we have provided the scores from the analysis of these transcriptions along with the inter-rater reliability data.

### Boundary extension test

The Boundary Extension Test that was used here differed from some previous versions, which showed only identical picture pairs on each trial^95^. Here eight additional picture pairs were included, four showing the second picture of the pair as further away, and four showing the second picture of the pair as closer up. However, the boundary extension effect is subtle, and by including pairs of pictures that were obviously different, the boundary extension effect may have been reduced. This can be observed in the responses made. In the current sample there were more “same” responses (49.90%) than “closer up” responses (42.26%), while previous studies report more “closer up” responses (around 60%) than “same” response (around 30%)^95^. Consequently, the boundary extension data are included here for completeness, but we advise caution in using and interpreting them.

### Supplementary information

Supplementary Tables 1-3

## Data Availability

No custom code was used in the processing of this dataset.
