# Supplementary material for: Release of cognitive and multimodal MRI data including real-world tasks and hippocampal subfield segmentations
Source: Sci Data. 2023 Aug 16;10:540. doi: 10.1038/s41597-023-02449-9 (PMC10432478; doi:10.1038/s41597-023-02449-9)
Supplement: Supplementary file 1 — Supplementary Tables 1-3 [file 41597_2023_2449_MOESM1_ESM.pdf]

Supplementary Information

Release of cognitive and multimodal MRI data including real-world tasks and hippocampal subfield segmentations

Ian A. Clark & Eleanor A. Maguire

Supplementary Table 1. The real-world tests and their associated measures.
pp. 2-3

Supplementary Table 2. The laboratory-based memory tests and their associated measures.
pp. 4-5

Supplementary Table 3. The laboratory-based general cognitive tests and their associated measures.
pp. 6-7

Supplementary Table 1. The real-world tests and their associated measures.

Main Measures	Sub-Measures
**Scene Construction Test**	
Experiential Index	
Content	Spatial References
	Entities Present
	Sensory Descriptions
	Thought/Emotions/Actions
Participant Ratings	Sense of Presence
	Vividness
	Difficulty
	Detail
	Memory Similarity
Spatial Coherence	Spatial Coherence Raw
	Spatial Coherence Normed
	Spatial Coherence Index
Experimenter Rating	Quality
**Autobiographical Interview**	
Internal Details	Internal Total
	Internal Events
	Internal Time
	Internal Place
	Internal Perceptual
	Internal Emotion
External Details	External Total
	External Event
	External Semantic
	External Repetition
	External Other
Experimenter Ratings	Episodic Richness
	Time
	Place
	Perceptual
	Emotion
	Time Integration
Participant Ratings	How clearly visualise
	Emotional change during event
	Importance of event now
	Importance of event then
	How often think about the event
**Future Thinking Test**	
Experiential Index	
Content	Spatial References
	Entities Present

	Sensory Descriptions
	Thought/Emotions/Actions
Participant Ratings	Sense of Presence
	Vividness
	Difficulty
	Detail
	Memory Similarity
Spatial Coherence	Spatial Coherence Raw
	Spatial Coherence Normed
	Spatial Coherence Index
Experimenter Rating	Quality
**Navigation Test**	
Overall Navigation Score	
Clip Recognition	
Scene Recognition	
Proximity Judgements	
Route Knowledge	
Sketch Map	Number of Road Segments
	Number of Road Junctions
	Number of Landmarks
	Landmark Placement
	Orientation Rating
	Overall Categorisation

Supplementary Table 2. The laboratory-based memory tests and their associated measures.

Laboratory-Based Memory Tests
**Rey–Osterrieth Complex Figure Test**
Copy Score
Delayed Recall Score
**Object-Place Association Test**
Trials to Criterion
Trial 1 Score
Trial 2 Score
Trial 3 Score
Trial 4 Score
Trial 5 Score
Trial 6 Score
Delayed Recall Score
**Rey Auditory Verbal Learning Test**
Trial 1 Score
Trial 2 Score
Trial 3 Score
Trial 4 Score
Trial 5 Score
Total Immediate Recall
List B Recall Score
Interference Recall Score
Delayed Recall Score
**Logical Memory Test**
Immediate Recall Raw Score
Immediate Recall Scaled Score
Delayed Recall Raw Score
Delayed Recall Scaled Score
**Wechsler Memory Scale Verbal Paired Associates Test**
Recall 1 Score
Recall 2 Score
Recall 3 Score
Recall 4 Score
Total Immediate Recall Raw
Immediate Recall Scaled
Delayed Recall Raw
Delayed Recall Scaled

Concrete Verbal Paired Associates Test

Recall 1 Score
Recall 2 Score
Recall 3 Score
Recall 4 Score
Total Immediate Recall Raw
Immediate Recall Scaled
Delayed Recall Raw
Delayed Recall Scaled

Abstract Verbal Paired Associates Test

Recall 1 Score
Recall 2 Score
Recall 3 Score
Recall 4 Score
Total Immediate Recall Raw
Immediate Recall Scaled
Delayed Recall Raw
Delayed Recall Scaled

Warrington Recognition Memory Tests for Words, Faces and Scenes

Words Raw
Words Scaled
Faces Raw
Faces Scaled
Scenes Raw*

Dead or Alive

Number Know
Number Correct
Proportion Correct

---

* Scaled scores are not available for this test.

Supplementary Table 3. The laboratory-based general cognitive tests and their associated measures.

Laboratory-Based General Cognitive Tests
**Test of Premorbid Functioning**
Raw Score
Estimate of the Full Scale Intelligence Quotient
**Matrix Reasoning Test**
Number Correct
Scaled Score
**Brixton Spatial Anticipation Test**
Raw Score
Scaled Score
**F-A-S Test**
Total Number of Words
**Digit Span Test**
Forwards Raw
Forwards Scaled
Backwards Raw
Backwards Scaled
**Symbol Span Test**
Raw Score
Scaled Score
**Paper Folding Test**
Number Correct
**Scene Description Test**
Spatial References
Entities Present
Sensory Descriptions
Thoughts/Emotions/Actions
Total Content
**Boundary Extension Test**
Percentage Much Closer
Percentage Little Closer

Percentage Same
Percentage Little Farther
Percentage Much Farther
Average Confidence Rating Much Closer
Average Confidence Rating Little Closer
Average Confidence Rating Same
Average Confidence Rating Little Farther
Average Confidence Rating Much Farther
Mean Score
